# Interaction Between Dendritic Cells and *Candida krusei* β-Glucan Partially Depends on Dectin-1 and It Promotes High IL-10 Production by T Cells

**DOI:** 10.3389/fcimb.2020.566661

**Published:** 2021-01-22

**Authors:** Truc Thi Huong Dinh, Phawida Tummamunkong, Panuwat Padungros, Pranpariya Ponpakdee, Lawan Boonprakong, Wilasinee Saisorn, Asada Leelahavanichkul, Patipark Kueanjinda, Patcharee Ritprajak

**Affiliations:** ^1^ Medical Microbiology Interdisciplinary Program, Graduate School, Chulalongkorn University, Bangkok, Thailand; ^2^ Research Unit in Integrative Immuno-Microbial Biochemistry and Bioresponsive Nanomaterials, Department of Microbiology, Faculty of Dentistry, Chulalongkorn University, Bangkok, Thailand; ^3^ Green Chemistry for Fine Chemical Productions STAR, Department of Chemistry, Faculty of Science, Chulalongkorn University, Bangkok, Thailand; ^4^ Oral Biology Research Center, Faculty of Dentistry, Chulalongkorn University, Bangkok, Thailand; ^5^ Translational Research in Inflammation and Immunology Research Unit (TRIRU), Department of Microbiology, Faculty of Medicine, Chulalongkorn University, Bangkok, Thailand; ^6^ Department of Microbiology, Faculty of Medicine, Chulalongkorn University, Bangkok, Thailand; ^7^ Department of Microbiology, Faculty of Dentistry, Chulalongkorn University, Bangkok, Thailand

**Keywords:** *Candida krusei*, β-glucan, dendritic cells, T cells, dectin-1, immune modulation

## Abstract

Host-*Candida* interaction has been broadly studied during *Candida albicans* infection, with a progressive shift in focus toward non-*albicans Candida* species. *C. krusei* is an emerging multidrug resistant pathogen causing rising morbidity and mortality worldwide. Therefore, understanding the interplay between the host immune system and *C. krusei* is critically important. *Candia* cell wall β-glucans play significant roles in the induction of host protective immune responses. However, it remains unclear how *C. krusei* β-glucan impacts dendritic cell (DC) responses. In this study, we investigated DC maturation and function in response to β-glucans isolated from the cell walls of *C. albicans*, *C. tropicalis*, and *C. krusei*. These three distinct *Candida* β-glucans had differential effects on expression of the DC marker, CD11c, and on DC maturation. Furthermore, bone-marrow derived DCs (BMDCs) showed enhanced cytokine responses characterized by substantial interleukin (IL)-10 production following *C. krusei* β-glucan stimulation. BMDCs stimulated with *C. krusei* β-glucan augmented IL-10 production by T cells in tandem with increased IL-10 production by BMDCs. Inhibition of dectin-1 ligation demonstrated that the interactions between dectin-1 on DCs and cell wall β-glucans varied depending on the *Candida* species. The effects of *C. krusei* β-glucan were partially dependent on dectin-1, and this dependence, in part, led to distinct DC responses. Our study provides new insights into immune regulation by *C. krusei* cell wall components. These data may be of use in the development of new clinical approaches for treatment of patients with *C. krusei* infection.

## Introduction

*Candida* species are the most common causative agents of opportunistic mycoses that impose increasing burdens of morbidity and mortality. In recent decades, mucocutaneous and invasive *Candida* infections caused by non-*albicans Candida* species have increased globally as a result of the development of anti-fungal drug resistance (Colombo et al., 2017; Kontoyiannis, 2017). *Candida krusei* commonly causes nosocomial infections in patients with hematologic malignancies (Kim et al., 2017; Lortholary et al., 2017; Jamiu et al., 2020), as well as osteomyelitis, pneumonia, vaginitis, endophthalmitis, endocarditis, oral candidiasis, and other conditions in patients with underlying medical complications (Jamiu et al., 2020). *C. krusei* is an emerging multi-drug resistant pathogen: it is intrinsically resistant to fluconazole and rapidly acquires resistance to other anti-fungal drugs such as flucytosine, amphotericin B and echinocandins (Jamiu et al., 2020). Hence, *C. krusei*-infected individuals have high mortality rates (Kronen et al., 2017; Jamiu et al., 2020).

Carbohydrate constituents of fungal cell walls are the main pathogen-associated molecular patterns (PAMPs) that trigger both host protective immunity and pathogenic inflammatory responses. β-glucan is one of the major carbohydrate components making up the inner skeleton of the *Candida* cell wall, and acts as a key PAMP triggering host immune responses (Gow et al., 2011; Netea et al., 2015). Recognition of β-glucan in the *Candida* cell wall by the dectin-1 receptor has been shown to play a key role in protective immunity and subsequent fungal eradication (Taylor et al., 2007; Gow et al., 2011). Moreover, levels of serum β-glucan shed from the cell wall were correlated with the clinical outcomes of patients with invasive candidiasis (Sims et al., 2012; Giacobbe et al., 2015). Therefore, circulating β-glucans may directly interact with immune cells and induce either protective immunity or pathologic inflammatory responses.

Dendritic cells (DCs) are antigen-presenting cells that play a key role in *Candida* recognition, phagocytosis, and killing (Newman and Holly, 2001; Netea et al., 2004). Interactions between invading fungi and DCs *via* pattern-recognition receptors (PRRs) such as C-type lectin receptors and Toll-like receptors (TLRs) allow DCs to develop functional versatility, which determines the fate of adaptive immune responses (Wuthrich et al., 2012). Engagement of dectin-1 on DCs leads to Syk activation and subsequent *C. albicans* clearance (Skrzypek et al., 2009). Dectin-1 is also required for DC discrimination of yeast and hyphae and to induce Th17-mediated anti-*Candida* immunity through an interleukin (IL)-6-dependent mechanism (Kashem et al., 2015). Furthermore, recent studies demonstrated that differential β-glucan exposure on the cell walls of various *Candida* species resulted in distinct immune responses (Chen et al., 2019; Thompson et al., 2019).

At present, little is known regarding the immune response to *C. krusei*, and the interplay between *C. krusei* β-glucan and DCs is poorly understood. In this study, we investigated the effects of *C. krusei*, *C. albicans*, and *C. tropicalis* β-glucans on DC activation and subsequent T cell responses. We also observed the differential dectin-1-mediated DC responses to the β-glucans of three distinct *Candida* species. Our data provide insights into β-glucan-DC interactions and subsequent regulation of T cell immunity.

## Materials and Methods

### Animals and Ethics Statement

Female C57BL/6s (5–6 weeks old) were purchased from Nomura Siam International Co., Ltd., Bangkok, Thailand. All animal procedures were performed in accordance with the guidelines and approved by the Chulalongkorn University Institutional Animal Care and Use Committee (IACUC) (Animal protocol 19-33-010 and 031/2561).

### *Candida* Strains and Culture

*C. albicans* strain SC5314 was used in this study as its cell wall β-glucans have been well characterized (Lowman et al., 2003a; Lowman et al., 2014). *C. tropicalis* strain ATCC 750 and *C. krusei* strain ATCC 6258 were selected because these reference strains are used for quality control and antifungal drug susceptibility testing. All *Candida* yeasts were grown in Yeast Peptone Dextrose (YPD) broth (HiMedia Laboratories, Mumbai, India) at 30°C for 6–8 h with 180 rpm shaking. Subsequently, the yeast cultures were diluted to an OD_600_ of 0.1 and grown in 1.2 L of YPD medium at 30°C for 13 h with 150 rpm shaking. Under these culture conditions, all *Candida* species grow as budding yeast-like cells (Katiyar and Edlind, 2001; Kadosh and Johnson, 2005; Suzuki et al., 2006). The morphologies of all *Candida* yeasts were assessed using bright field microscopy (Olympus BX50, Tokyo, Japan).

### Cell Wall β-Glucan Extraction

The protocols for *Candida* β-glucan extraction, depyrogenation and sterilization were kindly provided by East Tennessee State University, Johnson City, TN, USA. (Lowman et al., 2003a; Lowman et al., 2014). Briefly, *Candida* cell walls were first boiled at 100°C in 0.1 N NaOH for 15 min (three extractions). The harvested residues were next boiled at 100°C in 2 N H_3_PO_4_ for 15 min (three extractions), and the resulting residues were then boiled at 100°C in acidic ethanol (1% v/v of H_3_PO_4_ in absolute ethanol) for 15 min (three extractions) followed by neutralization to pH 7.0. The glucans were washed three times with endotoxin-free water. The glucans isolated using this procedure are water-insoluble microparticulates. Isolated β-glucans were depyrogenated in 250 mM NaOH and subsequently neutralized to pH 7.0 in 250 mM H_3_PO_4_. The β-glucans were washed in endotoxin-free water three times and sterilized by autoclaving at 121°C under 15 psi of pressure for 30 min. The amount of extracted β-glucans were used as dry weight. β-glucans were heat-dried at 50–60°C, and the dried glucans were weighted. The concentrations of β-glucans in all experiment are used as dry weight per volume. All yeast culture and β-glucan extraction procedures were performed using endotoxin free water and containers. The three batches of extracted glucans were pooled and used for all the experiments.

### Scanning Electron Microscopy

*Candida* β-glucans were fixed in 2.5% glutaraldehyde in 0.1 M phosphate buffer at pH 7.2 for 18 h at 4°C. The fixed β-glucans were washed with sterile deionized water and were air dried. The β-glucans were then processed for ultra-thin gold coating (JFC-1200, JEOL, Peabody, MA, USA). The specimens were observed by a scanning electron microscopy (Quanta250, FEI, Hillsboro, OR, USA) with 10,000x magnifications.

### Generation of Bone Marrow-Derived Dendritic Cells

DCs were isolated from murine bone marrow as described previously (Inaba et al., 2009; Nguyen et al., 2018). Briefly, bone marrow cells were isolated from femurs and tibias of mice. The cells (1×10^6^ per 1 ml) were seeded in 24-well plates and cultured in RPMI 1640 (GIBCO, Thermo Fisher Scientific, New York, NY, USA) containing 10% heat-inactivated fetal bovine serum (GIBCO), 0.2 mM Glutamax (GIBCO), 100 U/ml penicillin, 100 mg/ml streptomycin (GIBCO), 10 ng/ml recombinant murine granulocyte-macrophage colony-stimulating factor (Peprotech, Rocky Hill, NJ, USA), and 10 ng/ml recombinant murine IL-4 (Peprotech). The cells were incubated at 37°C under a humidified atmosphere containing 5% CO_2_ for 7 days. Half of the media volume was replaced every 2 days.

### Bone Marrow-Derived Dendritic Cell Stimulation With *Candida* β-Glucans

BMDCs were stimulated with β-glucans isolated from *C. albicans*, *C. tropicalis*, and *C. krusei* at concentrations of 12.5, 25, and 50 µg/ml for 24 h and 48 h. Unstimulated BMDCs were used as negative controls. Culture supernatants were collected for cytokine measurement and cells were harvested for evaluation of DC maturation marker expression.

### Dectin-1 Blockade

BMDCs were pre-treated with 25 µg/ml of a dectin-1 antagonist (soluble whole glucan particles; InvivoGen, San Diego, CA, USA) for 2 h and then 25 µg/ml of β-glucans isolated from *C. albicans*, *C. tropicalis*, and *C. krusei* were added to the culture. For comparison dectin-1 blockade, BMDCs were stimulated with 25 µg/ml of *Candida* β-glucans in the absence of dectin-1 antagonists. As a negative control, BMDCs were either untreated or pre-treated with dectin-1 antagonist. At 24 h and 48 h post-stimulation, culture supernatants were collected for cytokine measurement and cells were harvested for assessment of DC maturation marker expression.

### Syk Inhibition

BMDCs were pre-treated with 1 µM of Syk inhibitor (InvivoGen) for 30 min prior to treatment of 25 µg/ml of β-glucans isolated from *C. albicans*, *C. tropicalis*, and *C. krusei*. For comparison of dectin-1 blockade, BMDCs were stimulated with 25 µg/ml of *Candida* β-glucans in the absence of dectin-1 antagonists. As a negative control, BMDCs were either untreated or pre-treated with dectin-1 antagonist. At 24 h and 48 h post-stimulation, culture supernatants were collected for cytokine measurement and cells were harvested for assessment of DC maturation marker expression.

### Flow Cytometry Analysis

To evaluate BMDC maturation and inhibitory phenotype, BMDCs were stained with fluorophore-tagged monoclonal antibodies against mouse CD11c (N418), mouse CD40 (3/23), mouse CD80 (16-10A1), mouse CD86 (CL-1), mouse I-A/I-E (MHC class II) (M5/114.152), PD-L1 (MIH7), PD-L2 (TY25), CD206 (C068C2), and dectin-1 (RH1). For T cell assay, LN cells and splenocytes were stained with fluorophore-tagged monoclonal antibodies against mouse mouse CD3 (145-2C11), CD4 (GK1.5), mouse CD25 (PC61), and mouse/human FoxP3 (FJK-16s). Prior to staining with specific antibodies, the cells were incubated with Fc block (purified anti-mouse CD16/32) to reduce non-specific binding. All antibodies were obtained from BioLegend (San Diego, CA, USA) except for the anti-FoxP3 antibody, which was obtained from eBioscience (San Diego, CA, USA). The stained cells were assessed by flow cytometry (CytoFLEX, Beckman Coulter, San Diego, CA, USA) and the data were analyzed using Kaluza Flow Analysis Software (Beckman Coulter). For flow cytometry acquisition, the live cells of all samples were acquired at 20,000 cells/sample. Accordingly, the same electronic gate was applied to all sample for flow cytometry acquisition and analyses.

### *In Vitro* Dendritic Cell and T Cell Co-Culture Assay

BMDCs were stimulated with 25 µg/ml of *Candida* β-glucans for 24 h and the cells were then washed with RPMI medium twice. The BMDCs (1×10^5^ cells) were co-cultured with T cells (1×10^6^) isolated from spleens of intact mice using immunomagnetic beads (Pan T Cell Isolation Kit II, mouse; Miltenyi Biotec, San Diego, CA, USA) at a DC:T cell ratio of 1:10 in the presence of 30 ng/ml of soluble anti-CD3 antibody (145-2C11, Biolegend) in 48-well plates (Clement et al., 1985; Li and Kurlander, 2010). After 72 h, the culture supernatants were collected and the cells were stained for CD4, CD25 and FoxP3.

### *In Vivo* Immunization and *Ex Vivo* Re-Stimulation Assay

We performed subcutaneous immunization in the scruff of the neck with a mixture of 2 mg of *C. albicans* or *C. krusei* β-glucans and 30 μg of oval chicken albumin (OVA, Grade V, Sigma) in 200 μl PBS at day 0 and day 7. On day 14, draining lymph nodes (LNs) (cervical and brachial LNs) were excised from the mice and the LNs were digested with 300 units/ml Collagenase IV (GIBCO) and 20 units/ml DNase I (Sigma) at 37°C with 200 rpm shaking for 45 min. The LN cells were washed twice with RPMI containing 10% heat-inactivated FBS and pen/strep. The LNs were then seeded at 2 x 10^6^ cells in 500 μl of culture medium per well in 48-wells plate and stimulated with 200 μg/ml OVA. Culture supernatants were collected at 48 h post-stimulation for ELISA. The cells were harvested at 72 h post-stimulation and were then stained for CD3, CD4, CD25 and FoxP3.

### *In Vivo* Candida krusei Infection and *Ex Vivo* Re-Stimulation

The mice were divided into three groups (PBS, low dose, and high dose of *C. krusei*). Prior to *C. krusei* infection, dexamethasone (0.1 mg/g of body weight; Dexton-Vet, T.P. Drug laboratories (1969), Bangkok, Thailand) were intraperitoneally injected to the mice on day -3, -2, -1, and 5 to induced immunosuppression (Jacobsen et al., 2010). At day 0, mice were intravenously inoculated with 1×10^6^ and 5×10^6^ of *C. krusei* yeast cells in 100 ul of PBS. On day 7 post-infection, blood serum and spleens were harvested from the mice. Spleens were mechanically disrupted and treated with RBC lysis buffer (8.02 g NH4Cl, 0.84 g NaHCO3, and 0.37 g EDTA in 1 L sterile deionized water) to remove red blood cells. Splenocytes were then washed in RPMI medium containing 10% heat-inactivated FBS and pen/strep Then 2×10^6^ cells of splenocytes were stained for CD3, CD4, CD25, and FoxP3.

For *ex vivo* re-stimulation, purified anti-CD3 antibody (BioLegend, cat # 145-2C11) were immobilized on 24-well plat. Briefly, 10 μg/ml of purified anti-CD3 in 200 μl of Dulbecco’s Phosphate Buffered Saline (DPBS) were coated on the plate at 4°C overnight. Prior to cell seeding, the anti-CD3 solution were removed and the coated wells were rinsed with culture medium twice. Then, the splenocyted were seeded at the concentration of 4×10^6^ cells/well in 1 ml of culture medium (RPMI1640 supplemented with 10% of heat-inactivated FBS, 2 mM L-glutamax, 100 U/ml of penicillin, 100 U/mg of streptomycin, and 0.055 mM 2-mercaptoethanol). 48-h post-stimulation, culture supernatants were collected for cytokine measurements and the cells were harvested and stained for CD3, CD4, CD25, and FoxP3.

### Cytokine Quantification

Cytokines [interferon (IFN)-γ, IL-1β, IL-2, IL-6, IL-12, IL-17, IL-23, IL-10, and tumor necrosis factor (TNF)-α] in the culture supernatants of BMDCs and cytokines IFN-γ, IL-17, and IL-10 in the serum and culture supernatants of LN cells and splenocytes were quantified by sandwich ELISA following the manufacturer’s instructions. All ELISA kits were purchased from Biolegend, except the IL-23 ELISA kit, which was obtained from eBioscience. The absorbance was measured at 450 nm using a microplate reader (EPOCH2C, BioTek).

### Statistical Analysis

All data were presented as means ± standard deviations. Sample sizes are indicated in each figure legend. The data were analyzed using one-way ANOVA with post-hoc Tukey HSD test in SPSS 22 software (IBM, New York, NY, USA). Values of *p* < 0.05 were considered statistically significant.

## Results

### Morphology and Structure Analysis of *Candida* β-Glucans

**Figure 1:**
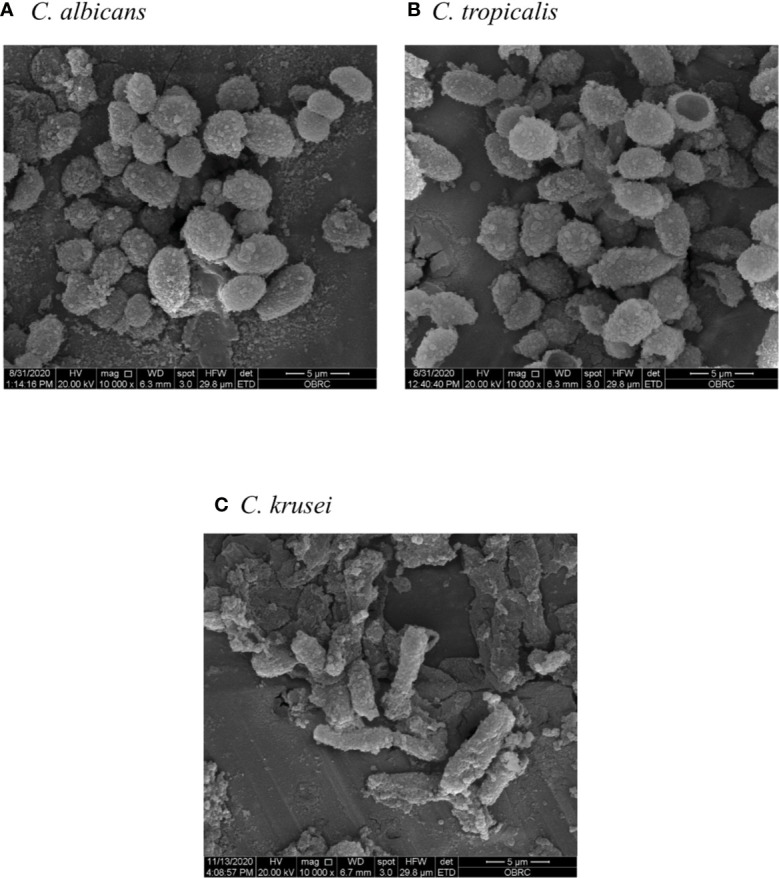
Morphology and size of *Candida* β-glucans. β-glucans of **(A)** *Candida albicans*, **(B)** *Candida tropicalis*, and **(C)** *Candida krusei* were observed by scanning electron microscopy (SEM) with 10,000x magnification.

First, β-glucans of *C. albicans, C. tropicalis*, and *C. krusei were* morphologically characterized by scanning electron microscopy (SEM) (**Figure 1**). β-glucans of *C. albicans* were similar to *C. tropicalis* in terms of size and shape (**Figures 1A, B**, respectively) The average size of *C. albicans* β-glucans was 4-5 µM and the average size of *C. tropicalis* β-glucans was 4–6 µM. Both *C. albicans* and *C. tropicalis* β-glucans formed oval shape. In addition, β-glucans of these two *Candida* species appeared as a dense fine speckled pattern. On the other hand, the morphology of *C. krusei* β-glucans was drastically different from the other two as they formed rod shape and large in size (average size is 7–10 µM), and surface of the β-glucans exhibited a course speckled pattern (**Figure 1C**).

**Figure 2:**
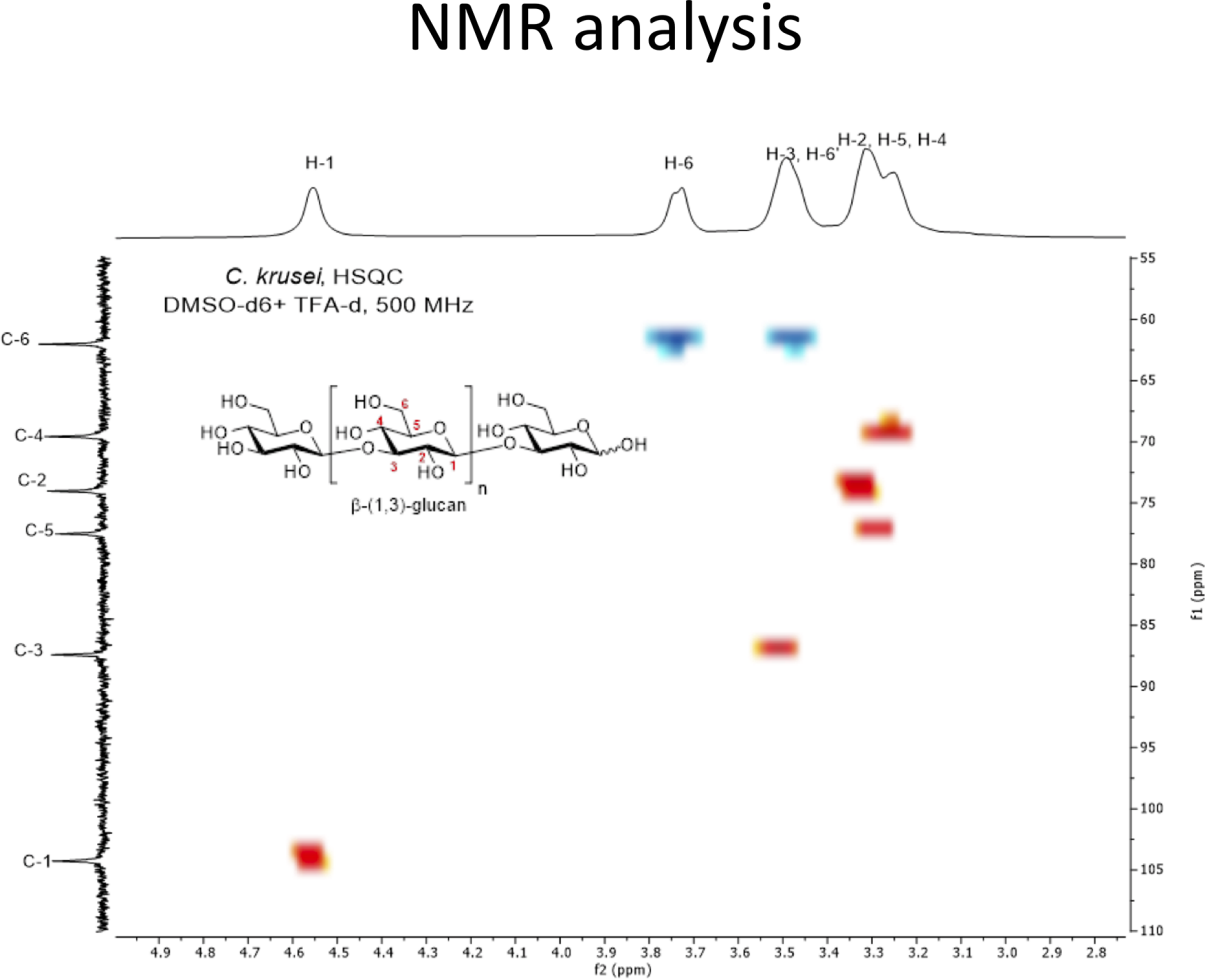
HSQC spectrum of *C. krusei* β-glucan (500 MHz, DMSO-d_6_ + TFA-d_1_). *C. krusei* β−glucan 50 mg was dissolved in DMSO-d_6_ (2.07 ml) and TFA-d_1_ (0.11 ml) then 0.5 mL of the solution was withdrawn to perform the HSQC experiment with 500 MHz NMR spectrometer at room temperature. The spectrum indicated a highly homogeneous β-(1,3)-glucan without β-(1,6)-branching.

In addition to morphological appearance, NMR analysis was performed to characterize *Candida* β-glucan structure. NMR characterizations of *Candida* β-glucans were challenging due to their low solubilities in all deuterated solvents (Padungros et al., 2012; Padungros et al., 2014). The addition of deuterated trifluoroacetic acid (TFA-d_1_) slightly enhanced the solubility. More importantly, TFA-d shifted all exchangeable protons on the hydroxyl groups of *Candida* β-glucans toward the downfield region, thus reducing overlapped protons signals on the pyranose rings. Characterization of *C. albicans* β-glucan and *C. tropicalis* β-glucan prove difficult due to their low solubilities in DMSO-d_6_ despite increasing temperature up to 60°C. Nevertheless, the ^1^H NMR spectra of both *C. albicans* β-glucan and *C. tropicalis* β-glucan were suggested that they were β-(1,3)-glucan with β-(1,6)-branching (**Supplementary Figure S1 and S2**) (Lowman et al., 2003b; Sukumaran et al., 2010; Lowman et al., 2014). For the *C. krusei* β-glucan, satisfying NMR spectra were obtained by performing measurement at room temperature. ^1^H NMR signals of anomeric proton (H-1) was identified at δ 4.56 ppm, H-6 was at δ 3.74 ppm, H-3/H-6’ were at δ 3.52–3.46 ppm, and H-2/H-5/H-4 were at δ 3.33–3.23 ppm (**Supplementary Figure S3**). These signals were corresponded to β-(1,3)-glucan structure. Surprisingly, characteristic β-(1,6)-glucan side chain signals at 4.24 ppm and 4.08 ppm were not observed (Kono et al., 2017). The simplicity of the ^1^H NMR signals indicated that the *C. krusei* β-glucan was a highly homogeneous β-(1,3)-glucan without β-(1,6)-branching. ^13^C NMR signals were also supported unbranched β-(1,3)-glucan structure and identified as following; β-anomeric carbon (C-1), C-3, C-5, C-2, C-4, and C-6 were at 104.3, 87.4, 77.5, 74.0, 69.6, and 62.0 ppm (**Supplementary Figure S4**) (Gerbst et al., 2013). The assignments of *C. krusei* β-glucan were further confirmed by COSY (**Supplementary Figure S5**) and HSQC (**Figure 2**) experiment which provided connectivity between ^1^H-C-C-^1^H (three-bond coupling) and ^1^H-^13^C (one-bond coupling) on the β-(1,3)-glucan, respectively.

### Effects of *Candida* β-glucans on Dendritic Cell Viability and CD11c Expression

To determine any potential contamination with bacterial endotoxin LPS during β-glucan preparation, we pre-treated our β-glucans with polymyxin B, a potent LPS neutralizer (Cardoso et al., 2007), before BMDC stimulation followed by an assessment of DC maturation marker expression (CD40, CD80, CD86, MHC class II) and inflammatory cytokine levels (IL-6 and TNF-α) (**Supplementary Figures S6-S7**). The results showed no significant difference in the expression of DC maturation markers and inflammatory cytokines between untreated and polymyxin B-treated groups, indicating that there was no endotoxin contamination in our β-glucans.

*Candida* β-glucans are PAMPs, which may transduce signals *via* PRRs and lead to cell death (Takeuchi and Akira, 2010; Kingeter and Lin, 2012). Therefore, we first examined DC viability in response to *Candida* β-glucan stimulation. BMDCs were stimulated with various concentrations of β-glucans isolated from the cell walls of *C. albicans*, *C. tropicalis*, and *C. krusei* for 24 h and 48 h, and the cell viability was determined by MTT assay (**Supplementary Figure S8**). At all doses tested, β-glucans from these three distinct *Candida* species showed no major effects on DC viability at both 24 h and 48 h (**Supplementary Figures S8A and S8B**, respectively) post-stimulation. The β-glucans of all *Candida* species had similar effects on DC viability. However, DC viability at 48 h showed a decreasing trend when BMDCs were stimulated with the highest concentration (50 µg/ml) of *C. albicans* and *C. krusei* β-glucans (**Supplementary Figure S8B**).

**Figure 3:**
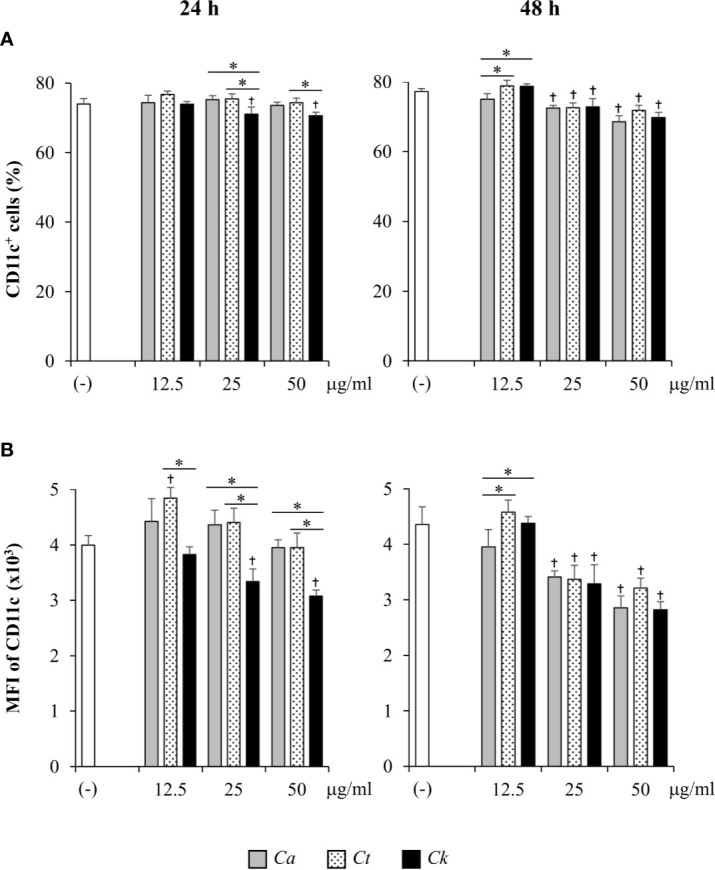
Determination of CD11c^+^ cell proportion and CD11c expression in bone-marrow derived dendritic cells (BMDCs) stimulated with *Candida* β-glucans. BMDCs were stimulated with 12.5, 25, and 50 µg/ml of β-glucans isolated from *C. albicans*, *C. tropicalis*, and *C. krusei* for 24 h and 48 h. Cells were analyzed by flow cytometry. **(A)** The percentages of CD11c^+^ cells were assessed using a dot pot analysis. **(B)** The geometric mean fluorescence intensity (MFI) of CD11c was determined using a histogram analysis. *n* = 5; data are representative of two independent experiments. ^†^ *p* < 0.05 compared with unstimulated BMDCs, *p < 0.05. (-), unstimulated BMDCs; *Ca*, *C. albicans*; *Ct*, *C. tropicalis*; *Ck*, *C. krusei*.

We next evaluated DC proportions in *Candida* β-glucan-stimulated bone marrow cells by flow cytometry analysis of the DC marker, CD11c (**Figure 3**). CD11c^+^ cells were gated and identified as DCs (**Supplementary Figures S9A and S10A**). At 24 h post-BMDC stimulation, all doses of *C. albicans* and *C. tropicalis* β-glucans did not alter the DC proportion, while *C. krusei* β-glucan at 25 and 50 µg/ml slightly reduced the percentage of CD11c^+^ cells compared with that among unstimulated BMDCs (**Figure 3A**, left panel). Furthermore, the proportions of CD11c^+^ cells among BMDCs stimulated with *C. krusei* β-glucan were significantly different from the proportions among BMDCs stimulated with *C. albicans* and *C. tropicalis* β-glucans (**Supplementary Figure S12A**, left panel). At 48 h post-stimulation, the percentages of CD11c^+^ cells among BMDCs stimulated with 25 and 50 µg/ml of all *Candida* β-glucans were reduced compared with unstimulated BMDCs (**Figure 3A**, right panel). However, a difference in DC proportion was not observed among BMDCs stimulated with different *Candida* β-glucans (**Supplementary Figure S12A**, right panel). CD11c expression levels of *Candida* β-glucan-stimulated BMDCs were also determined using histogram analyses of mean fluorescence intensity (MFI) (**Figure 3B** and **Supplementary Figure S11**). At 24 h post-stimulation, CD11c expression levels of BMDCs stimulated with *C. albicans* and *C. tropicalis* β-glucans were not clearly altered. By contrast, stimulation with *C. krusei* β-glucans (25 and 50 µg/ml) significantly diminished CD11c expression on BMDCs compared with expression on unstimulated BMDCs. CD11c expression in *C. krusei* β-glucan-stimulated BMDCs was markedly lower than that in *C. albicans* and *C. tropicalis* β-glucan-stimulated BMDCs (**Figure 3B**, left panel). At 48 h post-stimulation, the expression of CD11c was significantly reduced on BMDCs stimulated with 25 and 50 µg/ml of all *Candida* β-glucans. However, β-glucans of distinct *Candida* species showed no differential effect on CD11c expression (**Figure 3B**, right panel). Our data indicated that β-glucans from *C. albicans*, *C. tropicalis*, and *C. krusei* did not significantly affect DC viability and DC proportion. However, β-glucan from *C. krusei* downregulated CD11 expression on BMDCs.

### *Candida krusei* β-Glucans Had a Differential Impact on Dendritic Cell Maturation

Next, the effects of *Candida* β-glucans on DC maturation were evaluated. BMDCs were activated with various doses of *Candida* β-glucans for 24 h and 48 h, and expression of the DC maturation markers, CD40, CD80, CD86, and MHC class II, were determined by flow cytometry. First, the phenotypes of CD11c^+^ DCs were assessed using dot plot analyses (**Supplementary Figure S12**). The proportions of CD40^+^, CD80^+^, CD86^+^, and MHC class II^+^ subpopulations of CD11c^+^ cells were analyzed (**Supplementary Figure 9B and 10B**). BMDCs underwent maturation upon stimulation with all *Candida* β-glucans and the percentages of CD40^+^, CD80^+^, CD86^+^, and MHC class II^+^ cells were notably enhanced at both 24 h and 48 h post-stimulation (**Supplementary Figure S12**). BMDCs stimulated with three distinct *Candida* β-glucans showed similar proportions of CD40^+^ cells at both time points (**Supplementary Figure S12A**). At 24 h post-stimulation, the percentages of CD80^+^ cells (**Supplementary Figure S12B**, left panel) and MHC class II^+^ cells (**Supplementary Figure S12D**, left panel) among *C. krusei* β-glucan-stimulated BMDCs were significantly lower than those among *C. albicans* and *C. tropicalis* β-glucan-stimulated BMDCs. There were changes observed in the CD80^+^ subpopulation at 48 h post-stimulation: the proportion of CD80^+^ cells among all BMDCs was reduced, and the CD80^+^ cell proportion among BMDCs stimulated with *C. albicans* β-glucan was lower than that among BMDCs stimulated with *C. tropicalis* and *C. krusei* β-glucans (**Supplementary Figure S12B**, right panel). No difference in CD86^+^ cell proportion among stimulated BMDCs was observed at 24 h (**Supplementary Figure S12C**, left panel), but the CD86^+^ cell proportion among *C. krusei* β-glucan-stimulated BMDCs was higher than that among *C. albicans* and *C. tropicalis* β-glucan-stimulated BMDCs at 48 h post-stimulation (**Supplementary Figure S12C**, right panel). Although *Candida* β-glucans seemed to have differential effects on the proportions of mature DCs, only slight differences were observed.

**Figure 4:**
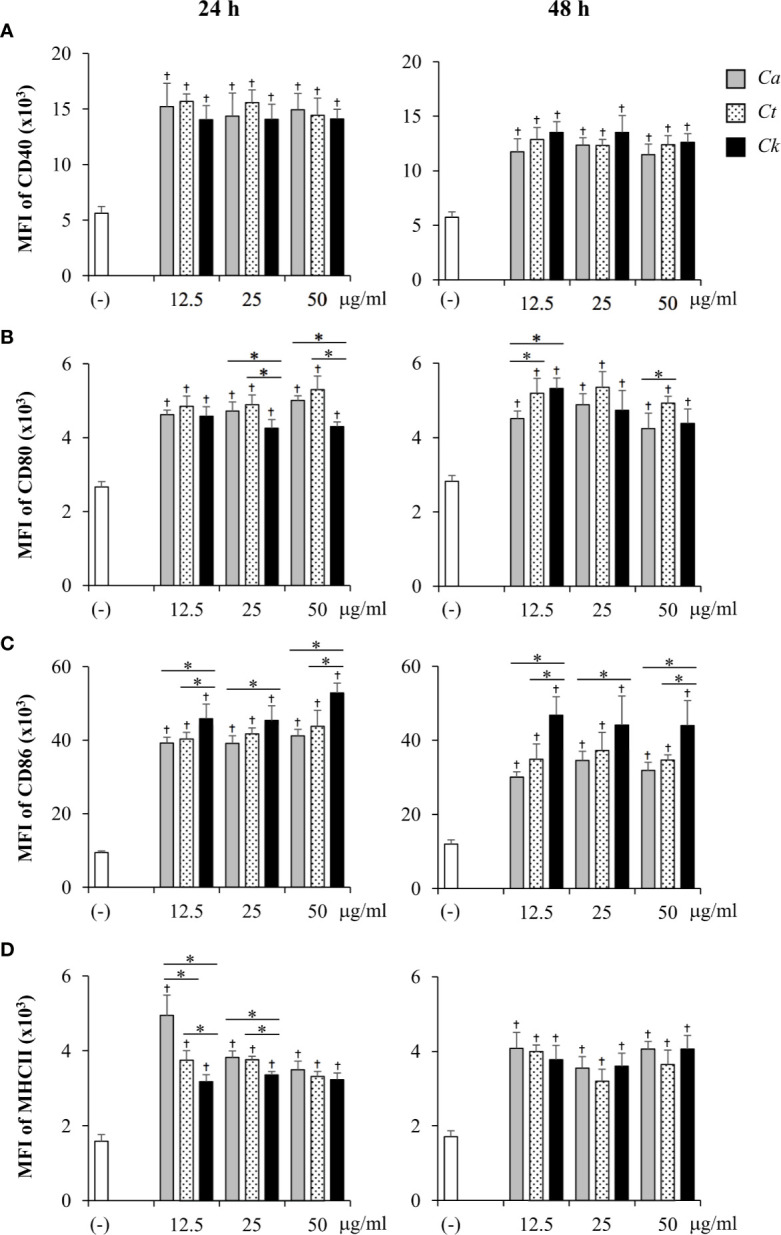
Differential DC maturation following stimulation with distinct *Candida* β-glucans. Bone-marrow derived dendritic cells (BMDCs) were stimulated with 12.5, 25, and 50 µg/ml of β-glucans isolated from *C. albicans*, *C. tropicalis*, and *C. krusei* for 24 h and 48 h, and the geometric mean fluorescence intensity (MFI) of dendritic cell (DC) maturation markers **(A)** CD40, **(B)** CD80, **(C)** CD86, and **(D)** MHC class II on CD11c^+^ cells were determined using histogram analyses. *n* = 5; data are representative of two independent experiments. ^†^ *p* < 0.05 compared with unstimulated BMDCs, *p < 0.05. (-), unstimulated BMDCs; *Ca*, *C. albicans*; *Ct*, *C. tropicalis*; *Ck*, *C. krusei*.

The expression levels of maturation markers on CD11c^+^ cells at 24 h and 48 h post-stimulation were also analyzed using histogram analyses of MFI values (**Supplementary Figures S13 and S14**). The effects of *Candida* β-glucans on expression levels of DC maturation markers were more apparent (**Figure 4**). Consistently, all markers were markedly upregulated on *Candida* β-glucan-stimulated BMDCs (**Figure 4**). The expression levels of CD40, CD80, and MHC class II on *Candida* β-glucan-stimulated BMDCs at both time points (**Figures 4A, B, D**) corresponded with the proportions of cells positive for these markers (**Figures 3A, B, D**). At 24 h post-stimulation, *C. krusei* β-glucan-stimulated BMDCs expressed lower levels of CD80 and MHC class II than *C. albicans* and *C. tropicalis* β-glucan-stimulated BMDCs (**Figures 4B, D**). By contrast, CD86 expression on *C. krusei* β-glucan-stimulated BMDCs was apparently higher than that on *C. albicans* and *C. tropicalis* β-glucan-stimulated BMDCs (**Figure 4C**).

Other DC markers, mannose receptor CD206, and programmed death-ligand 1 (PD-L1) and PD-L2, which play a role in DC immunomodulatory function (Serrano et al., 2018), were also examined in BMDCs stimulated with *Candida* β-glucans. Consistent to DC maturation markers, the expression levels of CD206, PD-L1, and PD-L2 in β-glucan-stimulated BMDCs were higher than in those expression in unstimulated group (**Supplementary Figures S13-S15**). However, there was no notable difference among *Candida* β-glucans on the expression of these molecules. Collectively, our findings suggested that cell wall β-glucans from distinct *Candida* species differentially affected DC phenotypes and maturation.

### *Candida krusei* β-Glucan Enhanced Pro-Inflammatory and Anti-Inflammatory Cytokine Production

**Figure 5:**
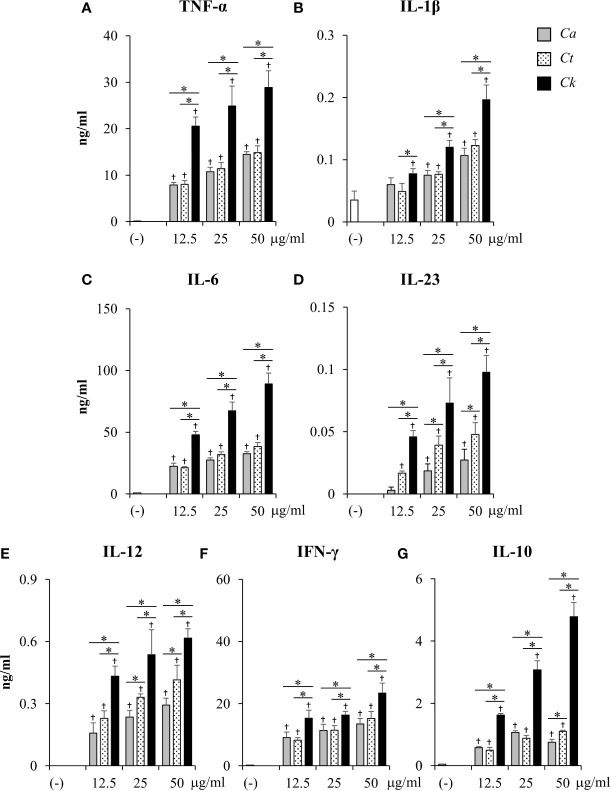
Excessive pro-inflammatory and anti-inflammatory cytokine production in *C. krusei* β-glucan-stimulated bone-marrow derived dendritic cells (BMDCs). BMDCs were stimulated with 12.5, 25, and 50 µg/ml of β-glucans isolated from *C. albicans*, *C. tropicalis*, and *C. krusei* for 24 h. Subsequently, the culture supernatants were collected and levels of **(A)** TNF-α, **(B)** IL-1β, **(C)** IL-6, **(D)** IL-23, **(E)** IL-12, **(F)** IFN-*γ*, and **(G)** IL-10 were quantitated by ELISA. *n* = 5; data are representative of two independent experiments. ^†^ *p* < 0.05 compared with unstimulated BMDCs, *p < 0.05. (-), unstimulated BMDCs; *Ca*, *C. albicans*; *Ct*, *C. tropicalis*; *Ck*, *C. krusei*.

To compare the effects of *Candida* β-glucans on DC cytokine production, the levels of pro-inflammatory cytokines (TNF-α, IL-1β, IL-6, IL-23, IFN-*γ*, IL-12) and anti-inflammatory cytokines (IL-10) were quantitated by ELISA (**Figure 5** and **Supplementary Figure S16**). At 24 h post-stimulation, all *Candida* β-glucans activated BMDCs to produce both pro-inflammatory and anti-inflammatory cytokines. Higher doses of *Candida* β-glucans induced more cytokine production (**Figure 5**). *C. albicans* and *C. tropicalis* β-glucans showed similar effects on the production of all cytokines. In contrast, *C. krusei* β-glucan augmented the production of all pro-inflammatory cytokines. Furthermore, levels of all pro-inflammatory cytokines induced by *C. krusei* β-glucan were approximately 0.5–2 fold higher than those induced by *C. albicans* and *C. tropicalis* β-glucans (**Figures 5A–F**). Intriguingly, *C. krusei* β-glucan had a major impact on IL-10 production: the amount of IL-10 produced by BMDCs stimulated with *C. krusei* β-glucan were approximately 3–5-fold greater than that produced by BMDCs stimulated with *C. albicans* and *C. tropicalis* β-glucans (**Figure 5G**). Cytokine production by *Candida* β-glucan-stimulated BMDCs was also determined at 48 h post-stimulation, and the results were consistent with the cytokine profiles at 24 h post-stimulation. Overall, levels of cytokines at 24 h and 48 h post-stimulation did not differ, except for a decline in IL-23 levels and a further increase in IFN-*γ* levels at 48 h post-stimulation (**Supplementary Figure S16**). Among *Candida* β-glucans, *C. krusei* β-glucan was the most potent stimulus of cytokine production by DCs, especially production of the anti-inflammatory cytokine, IL-10.

### *Candida krusei* β-Glucan-Stimulated BMDCs Promoted IL-10 Production in T Cells but Did Not Affect Regulatory T Cell Expansion Both *In Vitro* and *In Vivo*

**Figure 6:**
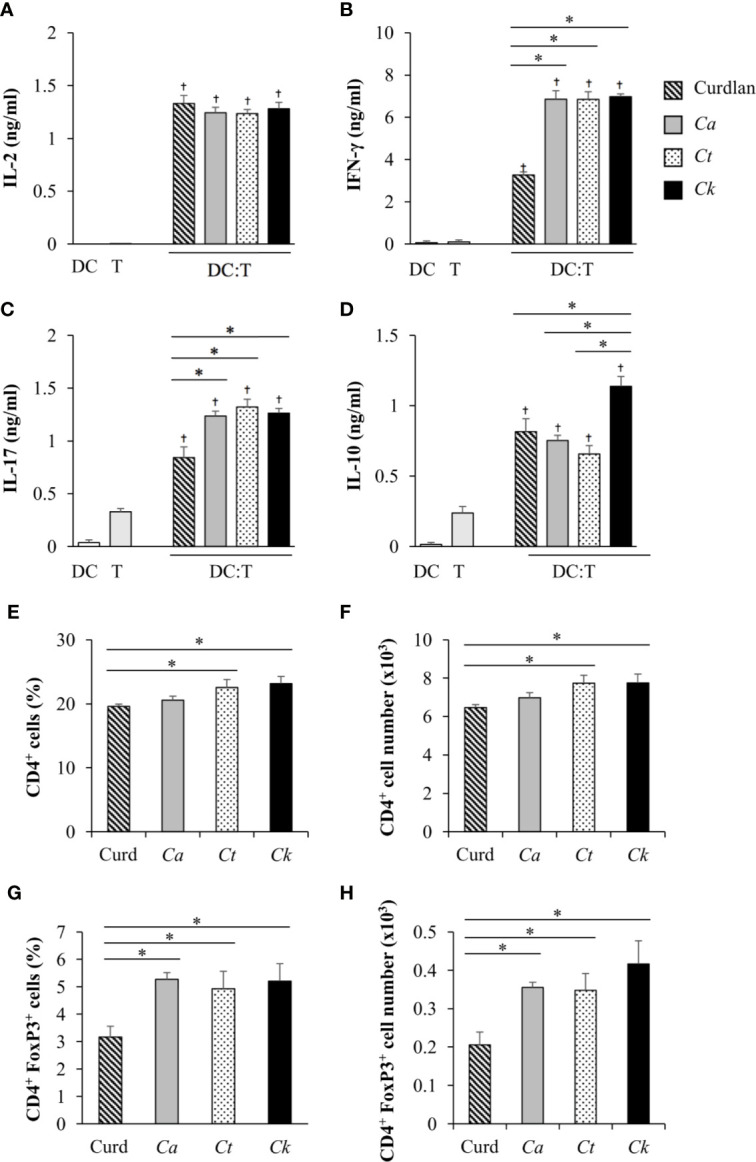
Effects of *C. krusei* β-glucans-stimulated bone-marrow derived dendritic cell (BMDC) on cytokine production of T cells. BMDCs were stimulated with 25 µg/ml of curdlan and β-glucans isolated from *C. albicans*, *C. tropicalis*, and *C. krusei* for 24 h. The stimulated BMDCs were co-cultured with murine splenic T cells at a DC:T cell ratio of 1:10 in the presence of soluble anti-mouse CD3 monoclonal antibody (30 ng/ml). Levels of T cell cytokines were assessed in culture supernatants. **(A)** IL-2 levels were measured following 24 h of co-culture. **(B)** IFN-*γ*, **(C)** IL-17, and **(D)** IL-10 levels were measured following 72 h of co-culture. BMDCs alone and T cells alone were incubated with the soluble anti-CD3 antibody as negative controls. *n* = 3; the percentages and numbers of CD4^+^ T cells and CD4^+^FoxP3^+^ were determined following 72 h of co-culture by flow cytometry. *n* = 3; **(E)** percent and **(F)** number of CD4^+^ cells, **(G)** percent, and **(H)** number of CD4^+^FoxP3^+^ cells. data are representative of two independent experiments. ^†^ *p*<0.05 compared with negative controls, *p<0.05. DC, BMDCs; T, T cells, *Ca*, *C. albicans*; *Ct*, *C. tropicalis*; *Ck*, *C. krusei*.

**Figure 7:**
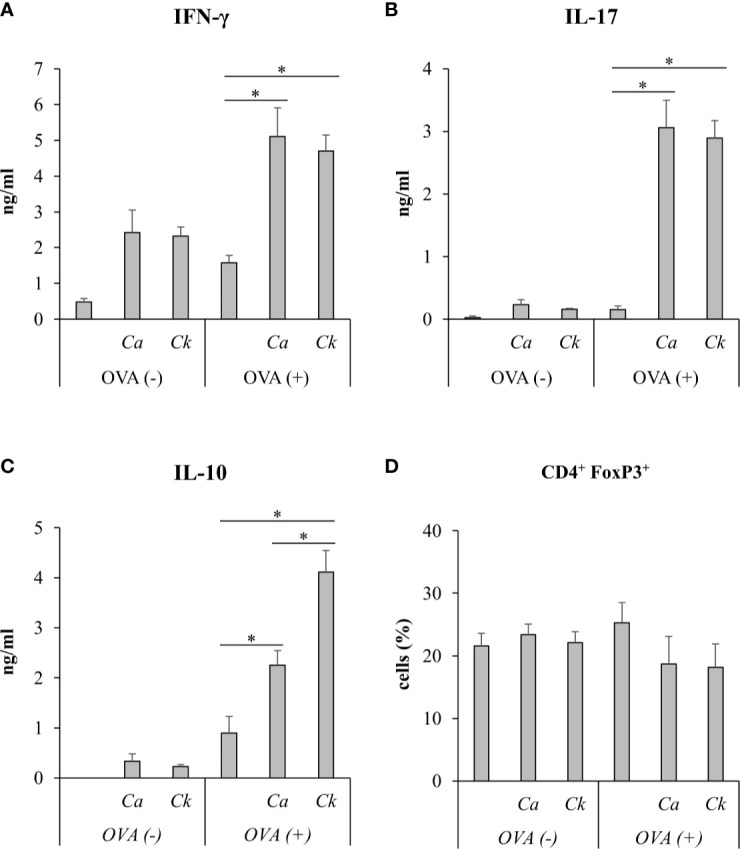
*In vivo* immunization of *C. albicans* or *C. krusei* β-glucans. Mice were subcutaneously immunized with a mixture of *C. albicans* or *C. krusei* β-glucans and OVA at day 0 and day 7. On day 14, the draining LNs cells were isolated and were *ex vivo* re-stimulated with OVA for 48 h. Culture supernatants were measured for **(A)** IFN-γ, **(B)** IL-17, and **(C)** IL-10 by ELISA. **(D)** CD4^+^FoxP3^+^ T cells were assessed by flow cytometry. *n* = 6. *p < 0.05. *Ca*, *C. albicans*; *Ck*, *C. krusei*.

Having demonstrated the effects of *Candida* β-glucans on DC maturation and cytokine production (**Figures 3**–**5**), we next examined the orchestration of T cell responses by *Candida* β-glucan-stimulated BMDCs. To assess the direct impact of DCs on T cell responses, we performed an *in vitro* DC-T co-culture experiment in the presence of a soluble anti-CD3 monoclonal antibody. In principle, the soluble anti-CD3 antibody binds to Fc receptors on DCs and is presented to T cells. Engagement of the soluble anti-CD3 antibody with T cells leads to transduction of the first signal into T cells (Clement et al., 1985; Li and Kurlander, 2010). Additional co-signals and cytokines were directly derived from *Candida* β-glucan-stimulated BMDCs. Using this approach, we investigated how DCs orchestrated T cell responses (**Figures 6** and **7**). BMDCs were stimulated with *Candida* β-glucans for 24 h and then co-cultured *in vitro* with T cells isolated from the spleens of mice in the presence of a low concentration (30 ng/ml) of soluble anti-mouse CD3 monoclonal antibody. Subsequently, levels of the T cell cytokines IL-2, IFN-*γ*, IL-17, and IL-10 were assessed by ELISA (**Figure 6**). As a negative control, DCs alone and T cells alone were also incubated with the soluble anti-CD3 antibody. A low dose of the soluble anti-CD3 antibody was unable to stimulate T cells as there was no production of a T cell proliferation factor, IL-2 (**Figure 6A**) (Spolski et al., 2018), and there was a low background of other cytokines (**Figures 6B–D**). Furthermore, no cytokines were released from BMDCs incubated with the soluble anti-CD3 antibody (**Figures 6A–D**). A commercial β-glucan, curdlan, was used as a positive control. BMDCs stimulated with curdlan and *Candida* β-glucans activated T cells in the presence of the soluble anti-CD3 antibody as shown by increased levels of IL-2. However, no difference in IL-2 levels was detected among the different β-glucans (**Figure 6A**). T cells co-cultured with *Candida* β-glucan-stimulated BMDCs secreted higher levels of IFN-*γ* and IL-17 than T cells co-cultured with curdlan-stimulated BMDCs (**Figures 6B, C**). We did not observe any differences in IFN-*γ* and IL-17 levels between BMDCs stimulated with the three distinct *Candida* β-glucans. *C. albicans* and *C. tropicalis* β-glucan-stimulated BMDCs drove T cells to produce IL-10 at similar levels to those produced by T cells activated with curdlan-stimulated BMDCs (**Figure 6D**). Strikingly, *C. krusei* β-glucan-stimulated BMDCs predominantly induced IL-10 secretion in T cells (**Figure 6D**); IL-10 production by T cells was correlated with the substantial IL-10 production by *C. krusei* β-glucan-stimulated BMDCs (**Figure 5G**).

IL-10-producing T cells possess regulatory function, and therefore we investigated whether FoxP3^+^ regulatory T cells were also affected by *C. krusei* β-glucan-stimulated BMDCs. In parallel to the observation of the T cell cytokine responses, the proportions and numbers of CD4^+^ T cells and CD4^+^FoxP3^+^ regulatory T cells were also determined. Following 72 h of DC-T cell co-culture in the presence of a soluble anti-CD3 antibody, CD4^+^ T cells were analyzed by flow cytometry (**Supplementary Figure S17** and **Figures 6E, F**). The proportions and numbers of CD4^+^ T cells among T cells co-cultured with *C. tropicalis* and *C. krusei* β-glucan-stimulated BMDCs were slightly higher than those among T cells co-cultured with curdlan and *C. albicans* β-glucan-stimulated BMDCs (**Figures 6E, F**). CD4^+^FoxP3^+^ regulatory T cell proportions and numbers among T cells activated with *Candida* β-glucan-stimulated BMDCs were significantly higher than those among T cells activated with curdlan-stimulated BMDCs (**Figures 6G, H**). However, BMDCs stimulated with *C. albicans*, *C. tropicalis*, and *C. krusei* had similar effects on CD4^+^FoxP3^+^ regulatory T cell expansion (**Figures 6G, H**).

To investigate the effect of *Candida* β-glucans on T cell response *in vivo*, we immunized mice with a mixture of OVA and *C. albicans* or *C. krusei* β-glucans subcutaneously because the resident DCs in the skin could be exposed to the stimuli (Nguyen et al., 2018). After immunization, the cells from draining LNs of the immunized mice were *ex vivo* re-stimulated with OVA and followed by cytokine production and CD4^+^FoxP3^+^ cell population analyses. There was no difference in the cytokines, IFN-γ and IL-17, production in T cells from the mice immunized with *C. krusei* β-glucans when compared to those cytokine productions T cells from the mice immunized with *C. albicans* β-glucans (**Figures 7A, B**). Consistent to our *in vitro* findings, *C. krusei* β-glucans substantially increased IL-10 production in T cells after OVA re-stimulation (**Figure 7C**), and there was no increase in CD4^+^FoxP3^+^ expansion in all groups (**Figure 7D**).

### Systemic Infection of *Candida krusei* Mediated High IL-10 Production in T Cells

**Figure 8:**
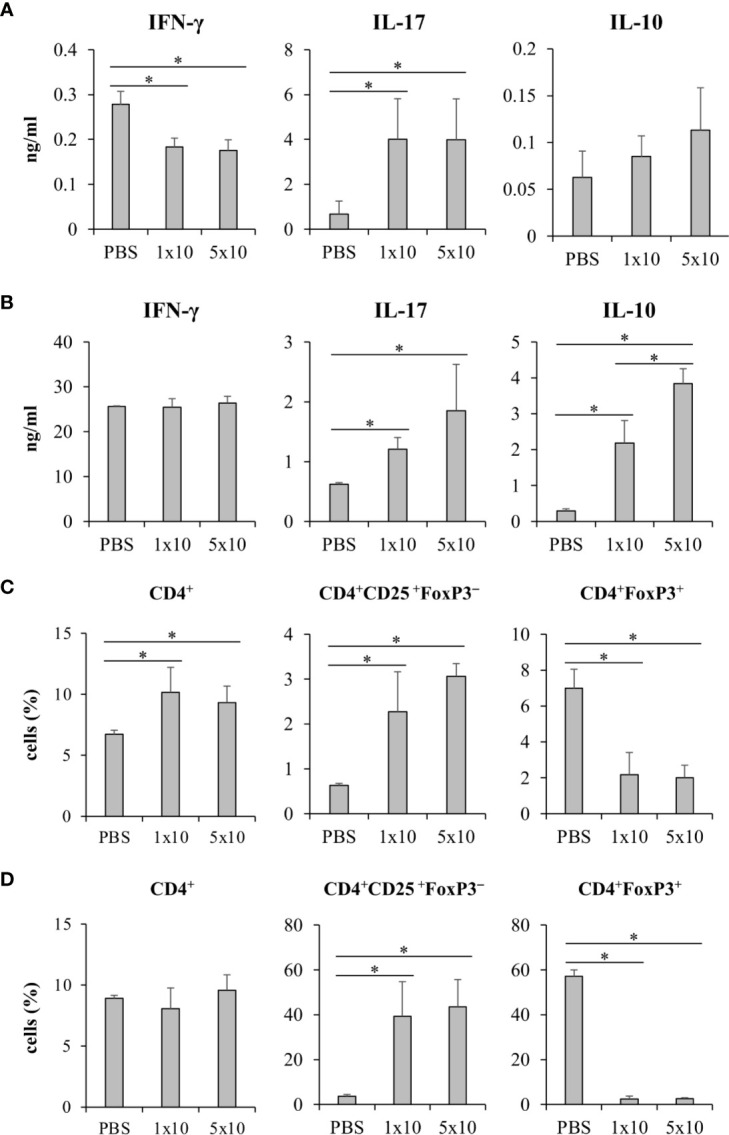
Systemic *C. krusei* infection promoted IL-10 production. Mice were treated with dexamethasone to induce immunosuppression and were systemically infected with 1x10^6^ and 5x10^6^ of *C. krusei*. Serum and spleens were collected on day 7 post-infection. Splenocytes were re-stimulated with immobilized anti-CD3 for 48 h. **(A)** Serum cytokines. **(B)** Cytokines from supernatant from anti-CD3-stimulated splenocytes. **(C)** T cells from unstimulated splenocytes. **(D)** T cells from anti-CD3 stimulated splenocytes. *n* = 5. *p < 0.05.

We next questioned whether *C. krusei* infection also mediates IL-10 production in T cells, therefore, *C. krusei* was systemically infected into mice. To recapitulate systemic candidiasis in immunocompromised patient, the mice were periodically treated with dexamethasone to induce immunosuppression (Jacobsen et al., 2010), and then followed by *C. krusei* infection. At day 7 post-infection, the sera were collected and the splenocytes were *ex vivo* re-stimulated with immobilized anti-mouse CD3 to specifically activate T cells (**Figure 8**). The blood sera exhibited substantial decrease in IFN-γ and increase in IL-17 levels but no alteration in IL-10 levels regardless of infection dose in *C. krusei*-infected group compared to the non-infected group (**Figure 8A**). The anti-CD3 re-activated splenocytes from *C. krusei* infected group did not show the change of IFN-γ production, while the cells released high amount of IL-17. However, there were no difference in IL-17 production in the re-activated splenocytes from low dose and high dose *C. krusei* infected mice (**Figure 8B**). Intriguingly, the production of IL-10 in the anti-CD3 re-activated splenocytes from *C. krusei* infected mice were obviously enhanced in a dose dependent manner (**Figure 8B**). The number of CD4^+^FoxP3^+^ cells did not expand but rather less than the control group after *C. krusei* infection in contrast to CD4^+^CD25^+^FoxP3^-^ population (**Figures 8C, D**). Thus, our results in systemic *C. krusei* infection were concordant with the observations in direct DC:T co-culture and *in vivo* immunization. Collectively, *C. krusei* β-glucan-stimulated BMDCs triggered substantial IL-10 production by T cells but did not enhance CD4^+^FoxP3^+^ regulatory T cell expansion.

### *Candida krusei* β-Glucan Interacted With Dectin-1 Receptor in a Different Manner From *C. albicans* and *C. tropicalis* β-Glucans

**Figure 9:**
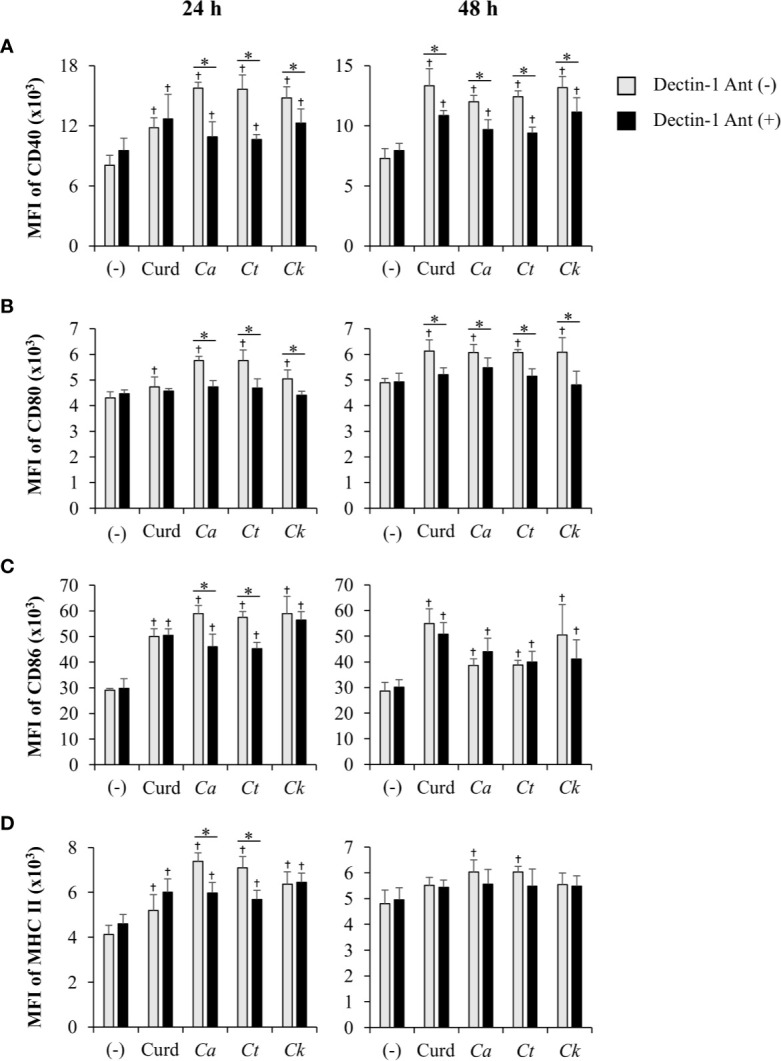
Inhibition of dendritic cell (DC) maturation by dectin-1 blockade. Bone-marrow derived DCs (BMDCs) were pre-treated with 25 µg/ml of dectin-1 antagonist for 2 h, and then the cells were stimulated with 25 µg/ml of curdlan and β-glucans isolated from *C. albicans*, *C. tropicalis*, and *C. krusei* for 24 h and 48 h. The geometric mean fluorescence intensity (MFI) of DC maturation markers **(A)** CD40, **(B)** CD80, **(C)** CD86, and **(D)** MHC class II on CD11c^+^ cells were determined using histogram analyses. *n* = 5; data are representative of two independent experiments. ^†^ *p* < 0.05 compared with unstimulated BMDCs, *p < 0.05. (-), unstimulated BMDCs; Curd, curdlan; *Ca*, *C. albicans*; *Ct*, *C. tropicalis*; *Ck*, *C. krusei*.

**Figure 10:**
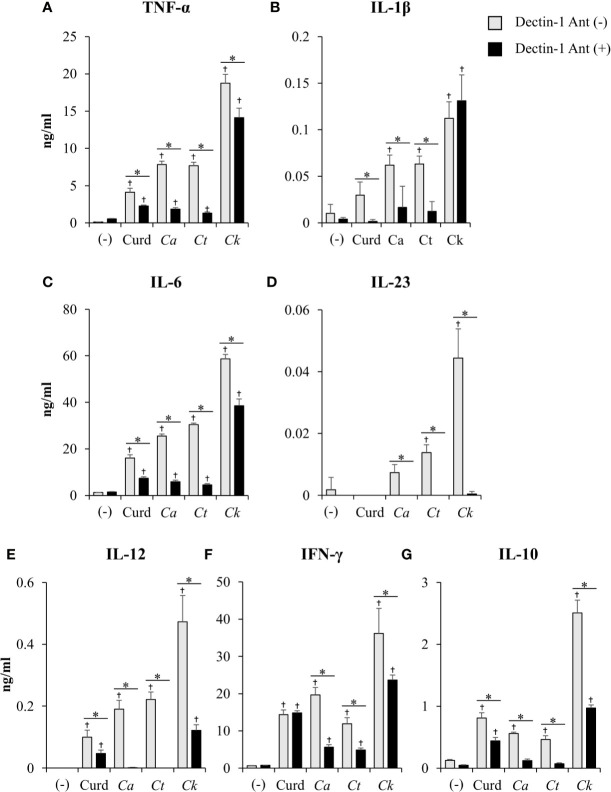
Contribution of dectin-1 blockade to dendritic cell (DC) cytokine production. Bone-marrow derived DCs (BMDCs) were pre-treated with 25 µg/ml of dectin-1 antagonist for 2 h, and then the cells were stimulated with 25 µg/ml of curdlan and β-glucans isolated from *C. albicans*, *C. tropicalis*, and *C. krusei* for 24 h. Levels of **(A)** TNF-α, **(B)** IL-1β, **(C)** IL-6, **(D)** IL-23, **(E)** IL-12, **(F)** IFN-*γ*, and **(G)** IL-10 were measured in culture supernatants by ELISA. *n* = 5; data are representative of two independent experiments. ^†^ *p*<0.05 compared with unstimulated BMDCs, *p<0.05. (-), unstimulated BMDCs; Curd, curdlan; *Ca*, *C. albicans*; *Ct*, *C. tropicalis*; *Ck*, *C. krusei*.

The dectin-1 receptor has been postulated to primarily recognize *Candida* β-glucans (Brown et al., 2003; Taylor et al., 2007), and its specific roles in recognition of β-glucans from different *Candida* species vary (Thompson et al., 2019). We therefore hypothesized that the differential responses of DCs to distinct *Candida* β-glucans may result, in part, from dissimilar interactions with dectin-1. To test this hypothesis, the dectin-1 receptor on DCs was blocked with a dectin-1 antagonist prior to *Candida* β-glucan stimulation, and then DC responses were assessed. Because curdlan is a well-known ligand of human and mouse dectin-1 receptor (Palma et al., 2006; Takano et al., 2017), it was used as the positive control in all dectin-1 blockade experiments. The DC proportion and CD11c expression in the dectin-1 blocking assay were first assessed by flow cytometry (**Supplementary Figure S18**). Blockade of dectin-1 did not affect DC proportion or CD11c expression in BMDCs stimulated with curdlan and all *Candida* β-glucans (**Supplementary Figures S18A and S18B**). Next, we examined the effect of dectin-1 blockade on DC maturation in response to *Candida* β-glucan stimulation. We assessed expression levels (MFI) of DC maturation markers at 24 h and 48 h post-stimulation (**Supplementary Figures S19 and S20**, respectively, and **Figure 9**). Dectin-1 antagonism efficiently reduced CD40 and CD80 expression on BMDCs stimulated with curdlan and all *Candida* β-glucans (**Figures 9A, B**). Blockade of dectin-1 inhibited the expression of CD86 and MHC class II on BMDCs stimulated with *C. albicans* and *C. tropicalis* β-glucans, but not on BMDCs stimulated with curdlan and *C. krusei* β-glucan (**Figures 9C, D**). Alterations of pro-inflammatory and anti-inflammatory cytokine responses following dectin-1 blockade were also investigated (**Figure 10** and **Supplementary Figure S21**). Overall, inhibition of dectin-1 receptor ligation dampened production of all cytokines in curdlan- and *Candida* β-glucan-stimulated BMDCs at 24 h (**Figures 10A–G**) and 48 h (**Supplementary Figure S21A–G**).

To assess variation in the interactions between the three *Candida* β-glucans and dectin-1, we further analyzed the percent blocking of expression of DC maturation markers (**Table 1**) and levels of DC cytokines (**Table 2**). Dectin-1 antagonism less potently inhibited DC maturation in response to *C. krusei* β-glucan, especially at 24 h post-stimulation (**Table 1**), and least efficient blocked DC cytokine production in response to *C. krusei* β-glucan (**Table 2**). These results suggested that the interaction between DCs and *C. krusei* β-glucan may be partially dependent on dectin-1. In addition, the percent blocking of production of some cytokines in *C. albicans* and *C. tropicalis* β-glucan-stimulated BMDCs differed.

**Table 1:** Blockade of dendritic cell (DC) maturation by dectin-1 antagonist.

		% Blocking* ± SD			
		Curdlan	*C. albicans*	*C. tropicalis*	*C. krusei*
**24 h**	**CD40**	2.87 ± 3.98^b,c,d^	30.86 ± 9.54 ^a,d^	32.21 ± 3.27 ^a,d^	17.05 ± 9.65 ^a,b,c^
	**CD80**	3.53 ± 2.01^b,c^	17.89 ± 4.32^a^	18.61 ± 6.14^a^	12.56 ± 7.07
	**CD86**	1.58 ± 2.27^b,c,d^	21.77 ± 8.28 ^a,d^	21.19 ± 4.29 ^a,d^	4.53 ± 5.30 ^a,b,c^
	**MHC class II**	0.00 ± 0.00^b,c,d^	18.92 ± 6.22 ^a,d^	25.77 ± 5.32 ^a,d^	2.00 ± 2.74 ^a,b,c^
**48 h**	**CD40**	18.52 ± 3.20	19.42 ± 6.89	24.32 ± 4.00	15.62 ± 9.16
	**CD80**	15.03 ± 4.22	9.58 ± 6.27	15.19 ± 4.78	21.16 ± 10.52
	**CD86**	6.13 ± 7.74	1.12 ± 2.51^d^	2.09 ± 2.43^d^	18.57 ± 14.82^b,c^
	**MHC class II**	2.72 ± 2.86	7.84 ± 9.18	10.75 ± 7.97	3.78 ± 3.54

*% Blocking was calculated by the following formula; (average MFI of molecule A expressed on glucan-stimulated DCs – MFI of molecule A expressed on dectin-1 antagonist treated glucan-stimulated DCs) x 100/average MFI of molecule A expressed on glucan-stimulated DCs). n = 5; Data are representatives of two independent experiments. ^a^p < 0.05 compared with Curdlan, ^b^p < 0.05 compared with C. albicans glucan, ^c^p < 0.05 compared with C. tropicalis glucan, ^c^p < 0.05 compared with C. krusei glucan. ^d^p < 0.05 compared with C. krusei glucan.

**Table 2:** Blockade of dendritic cell (DC) cytokine production by dectin-1 antagonist.

		% Blocking* ± SD			
		Curdlan	*C. albicans*	*C. topicalis*	*C. krusei*
**24 h**	**TNF-α**	45.14 ± 3.39^b,c,d^	76.37 ± 2.53^a,d^	83.12 ± 2.22^a,d^	24.70 ± 6.89^a,b,c^
	**IL-1β**	92.19 ± 17.47^d^	73.30 ± 36.88 ^d^	80.77 ± 16.87 ^d^	4.02 ± 8.98^a,b,c^
	**IL-6**	54.53 ± 4.55^b,c,d^	76.98 ± 2.60^a,c,d^	85.22 ± 1.95^a,b,d^	34.18 ± 4.90^a,b,c^
	**IL-23**	N.D.	100.00 ± 0.00	100.00 ± 0.00	99.12 ± 1.96
	**IL-12**	53.02 ± 10.84^b,c,d^	99.58 ± 0.94^a,d^	100.00 ± 0.00^a,d^	74.25 ± 3.86^a,b,c^
	**IFN-γ**	0.29 ± 0.64^b,c,d^	71.53 ± 3.53^a,c,d^	59.43 ± 4.53^a,b,d^	34.55 ± 3.75^a,b,c^
	**IL-10**	45.89 ± 7.27^b,c,d^	78.42 ± 4.69^a,d^	84.37 ± 2.67^a,d^	61.26 ± 1.98^a,b,c^
**48 h**	**TNF-α**	42.62 ± 6.14^b,c,d^	74.39 ± 1.47^a,d^	81.50 ± 0.89^a,d^	28.92 ± 8.57^a,b,c^
	**IL-1β**	100.00 ± 0.00^d^	100.00 ± 0.00 ^d^	100.00 ± 0.00 ^d^	59.55 ± 9.40^a,b,c^
	**IL-6**	51.43 ± 2.61^b,c,d^	71.45 ± 1.76^a,c,d^	79.72 ± 1.70^a,b,d^	40.52 ± 4.67^a,b,c^
	**IL-23**	N.D.	100.00 ± 0.00	100.00 ± 0.00	99.75 ± 0.57
	**IL-12**	63.96 ± 7.89^b,c,d^	100.00 ± 0.00^a,d^	100.00 ± 0.00^a,d^	75.88 ± 4.91^a,b,c^
	**IFN-γ**	30.30 ± 6.18^b,c^	68.77 ± 1.91^a,d^	66.84 ± 1.53^a,d^	18.18 ± 11.75 ^b,c^
	**IL-10**	48.63 ± 7.54^b,c,d^	79.60 ± 4.24^a,d^	84.57 ± 3.74^a,d^	60.02 ± 4.36^a,b,c^

*% Blocking was calculated by the following formula; (average MFI of cytokine A produced by glucan-stimulated DCs – MFI of cytokine A produced by dectin-1 antagonist treated glucan-stimulated DCs) x 100/average MFI of cytokine A produced by glucan-stimulated DCs). n = 5; Data are representatives of two independent experiments. ^a^p < 0.05 compared with Curdlan, ^b^p < 0.05 compared with C. albicans glucan, ^c^p < 0.05 compared with C. tropicalis glucan, ^c^p < 0.05 compared with C. krusei glucan. ^d^p < 0.05 compared with C. krusei glucan.

**Figure 11:**
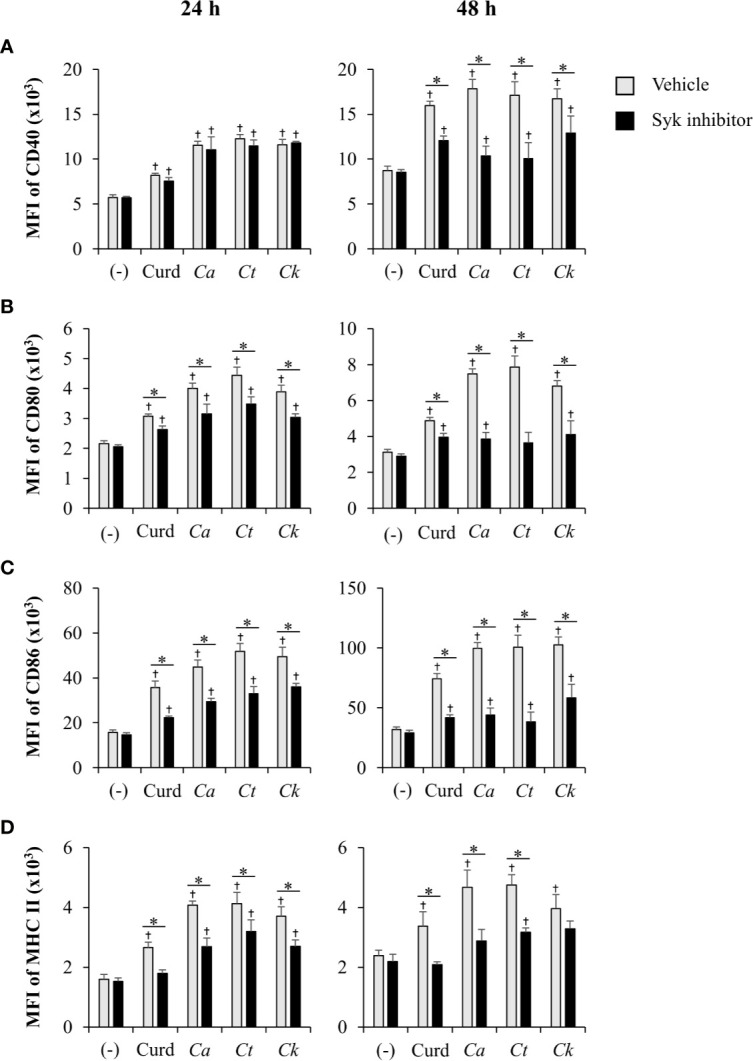
Inhibition of dendritic cell (DC) maturation by Syk inhibitor. Bone-marrow derived DCs (BMDCs) were pre-treated with 1 µM of Syk inhibitor for 30 min, and then the cells were stimulated with 25 µg/ml of curdlan and β-glucans isolated from *C. albicans*, *C. tropicalis*, and *C. krusei* for 24 h and 48 h. The geometric mean fluorescence intensity (MFI) of DC maturation markers **(A)** CD40, **(B)** CD80, **(C)** CD86, and **(D)** MHC class II on CD11c^+^ cells were determined using histogram analyses. *n* = 5; data are representative of two independent experiments. ^†^ *p* < 0.05 compared with unstimulated BMDCs, *p < 0.05. (-), unstimulated BMDCs; Curd, curdlan; *Ca*, *C. albicans*; *Ct*, *C. tropicalis*; *Ck*, *C. krusei*.

**Figure 12:**
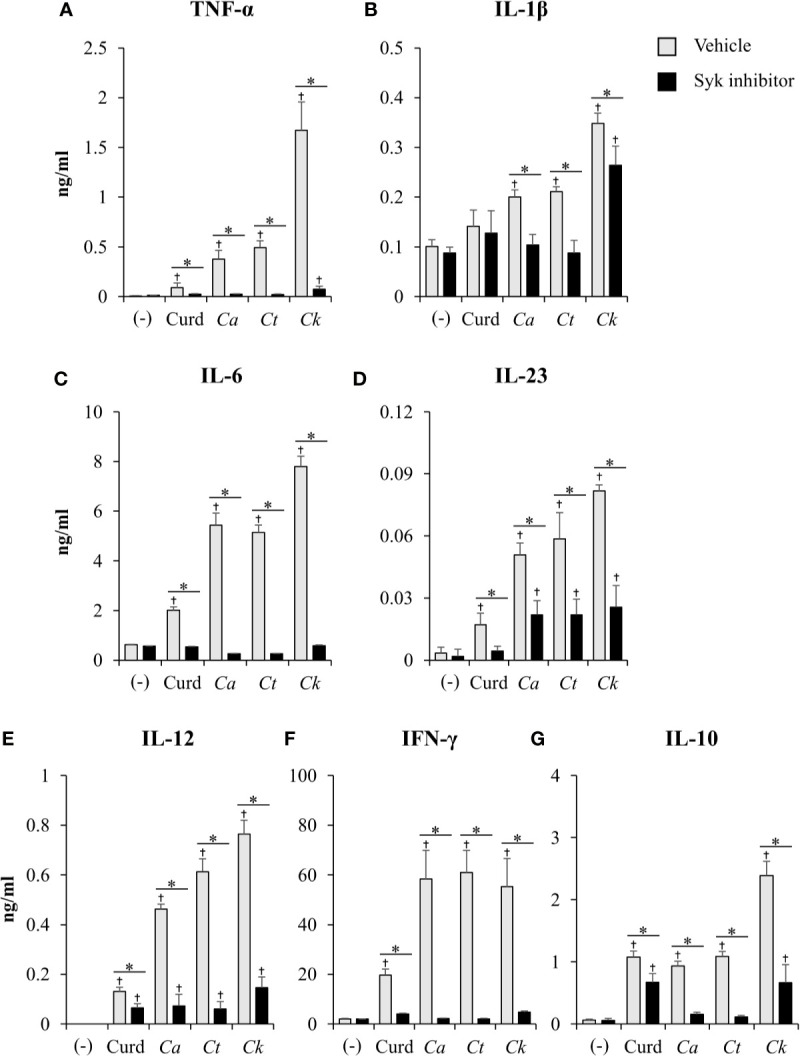
Effects of Syk inhibition on dendritic cell (DC) cytokine production. Bone-marrow derived DCs (BMDCs) were pre-treated with 1 µM of Syk inhibitor for 30 min, and then the cells were stimulated with 25 µg/ml of curdlan and β-glucans isolated from *C. albicans*, *C. tropicalis*, and *C. krusei* for 48 h. Levels of **(A)** TNF-α, **(B)** IL-1β, **(C)** IL-6, **(D)** IL-23, **(E)** IL-12, **(F)** IFN-*γ*, and **(G)** IL-10 were measured in culture supernatants by ELISA. *n* = 5; data are representative of two independent experiments. ^†^ *p* < 0.05 compared with unstimulated BMDCs, *p < 0.05. (-), unstimulated BMDCs; Curd, curdlan; *Ca*, *C. albicans*; *Ct*, *C. tropicalis*; *Ck*, *C. krusei*.

Coupled to dectin-1, spleen tyrosine kinase (Syk) has been well described as a crucial adaptor protein kinase of signaling pathway downstream of dectin-1 in DC anti-fungal mechanism (Whitney et al., 2014). We then investigated whether different *Candida* β-glucans also affect DC functions through Syk signaling pathway. BMDCs were treated with Syk inhibitor prior to *Candida* β-glucan stimulation. At 24 h and 48 h post-stimulation, DC maturation and cytokine production were observed (**Figures 11** and **12**, **Supplementary Figure S22**, **Supplementary Tables S1 and S2**). Syk inhibitor could suppress DC maturation as indicated by significant reduction of CD40, CD80, CD86, and MHC class II expression as early as 24 h post-stimulation (**Figure 11**). Expectedly, inhibition of Syk also diminished the cytokines (TNF-α, IL-1β, IL-6, IL-23, IL-12, IFN-γ, and IL-10) released by BMDCs stimulated with different *Candida* β-glucans (**Figure 12** and **Supplementary Figure S22**). The data of Syk inhibition also showed the differential activation of BMDCs by the distinct *Candida* β-glucans (**Supplementary Tables S1 and S2**).

**Figure 13:**
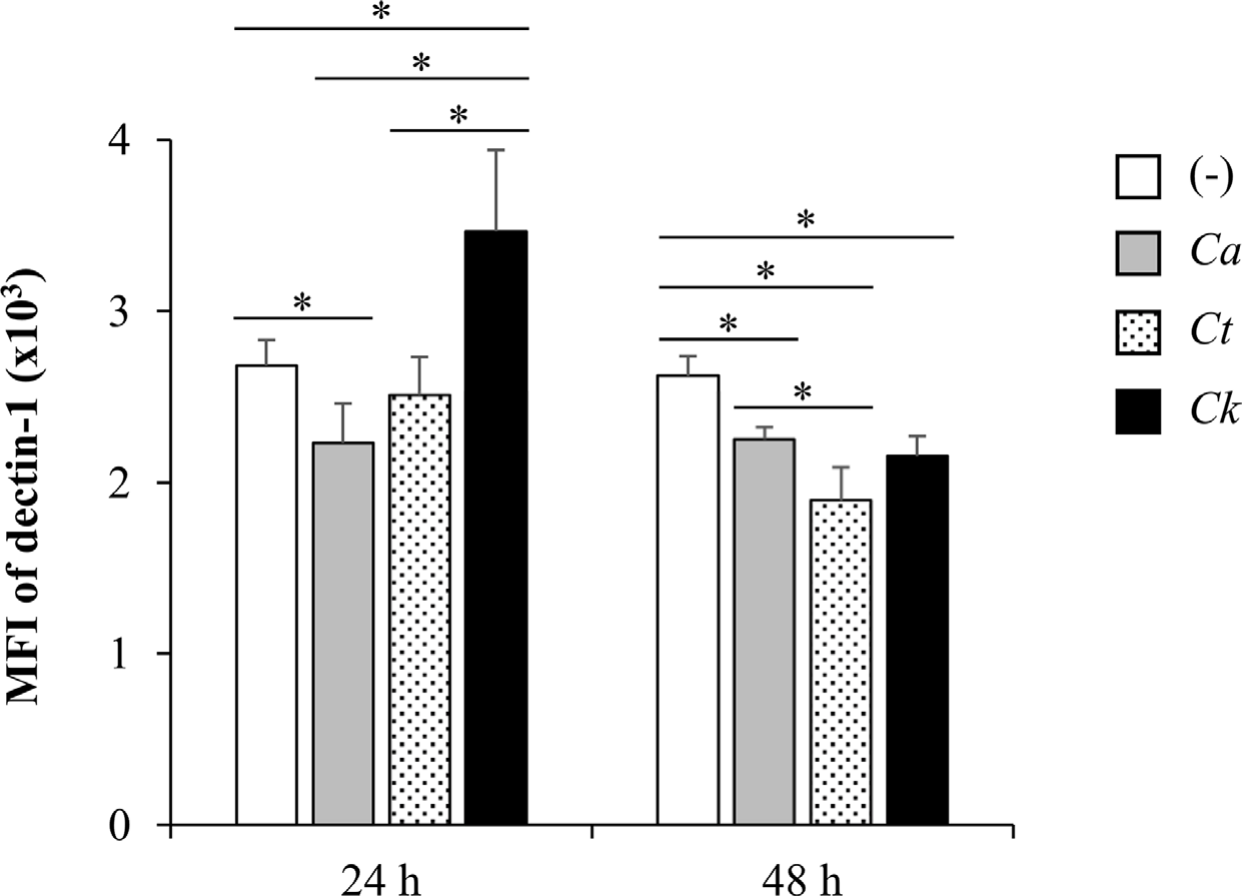
Differential dectin-1 expression on Candida β-glucan-stimulated bone-marrow derived dendritic cells (BMDCs). BMDCs were stimulated with 25 µg/ml of β-glucans isolated from *C. albicans*, *C. tropicalis*, and *C. krusei* for 24 h and 48 h, and the geometric mean fluorescence intensity (MFI) of dectin-1 on CD11c^+^ cells were determined using histogram analyses. *n* = 5; data are representative of two independent experiments. *p < 0.05. (-), unstimulated BMDCs; *Ca*, *C. albicans*; *Ct*, *C. tropicalis*; *Ck*, *C. krusei*.

Having found that *Candida* β-glucans and dectin-1 interactions regulated Syk-mediated cytokine production, we next questioned whether these β-glucans affect the expression of dectin-1 receptor on the DCs. To investigate this, we analyzed surface dectin-1 expression on CD11c^+^ BMDCs after *Candida* β-glucan stimulation (**Figure 13** and **Supplementary Figure S23**). *C. albicans* β-glucan downmodulated dectin-1 expression on BMDCs at 24 h and 48 h post-stimulation while *C. tropicalis* β-glucan did no change level of dectin-1 on BMDCs at 24 h but rather downmodulated the dectin-1 expression at 48 h. Intriguingly, dectin-1 expression on BMDCs stimulation with *C. krusei* β-glucans was significantly enhanced at 24 h, and it was downmodulated at 48 h (**Figure 13**).

Collectively, these findings suggested the involvement of dectin-1 receptor and Syk signaling pathway in DC maturation and cytokine production in response to *C. albicans*, *C. tropicalis*, and *C. krusei* β-glucans, and suggested that the differential DC responses to distinct *Candida* β-glucans possibly resulted from dectin-1 expression and these differential interactions of the *Candida* β-glucans with dectin-1 were through dectin-1/Syk signaling pathway axis.

## Discussion

Cell wall β-glucans of *C. albicans* have been demonstrated to play a pivotal role in the induction of anti-fungal immunity (Netea et al., 2015; Camilli et al., 2018). However, our findings demonstrated that the cell wall β-glucans of *C. albicans*, *C. tropicalis*, and *C. krusei* have different impacts on DC immunity, which consequently influenced T cell responses. Most *Candida* species can undergo a yeast-to-hypha transition in response to environmental conditions such as high temperature, pH, and nutritional factors (Thompson et al., 2011; Lu et al., 2014). β-glucan is a major carbohydrate component of the *Candida* cell wall, located in the intermediate layer between mannan and chitin, and is exposed on the outer surface (Wheeler et al., 2008; Gow et al., 2011). β-glucan is masked by mannan in the cell wall of *C. albicans* hyphae (Bain et al., 2014; Davis et al., 2014). The yeast form in all *Candida* species plays a key role in the early stages of host tissue colonization (Netea et al., 2015). For these reasons, we explored the role of β-glucans in yeast cell walls, which may be linked to the physiological and immunological responses occurring in *Candida*-infected individuals.

Our investigations demonstrated quantitative differences in DC maturation and function in response to the cell wall β-glucans of three distinct *Candida* species (**Figures 3**–**5**). Several lines of evidence suggest that the differential effects of *Candida* β-glucans are probably related to variation in β-glucan structures. The different β-glucan structures in yeast and hyphae of *C. albicans* resulted in distinct cytokine responses in human monocyte-derived macrophages (Lowman et al., 2014). In addition, the different β-glucan contents of two distinct strains of *C. tropicalis* differentially elicited immune responses from peripheral blood mononuclear cells (Mesa-Arango et al., 2016). Although there is no direct evidence that the β-glucan structures of *C. tropicalis* and *C. krusei* differ, detection of β-glucans in the cell lysates of *Candida* species using β-glucan-specific antibodies reflected the inter-species diversity of β-1,6-glucan and β-1,6-glucan contents and structures (Matveev et al., 2019; Yamanaka et al., 2020). Consistently, our NMR analysis revealed the distinct β-glucan structure of *C. krusei* which mainly contained β-1,3-glucan (**Figure 2**). Meanwhile, *C. albicans* and *C. tropicalis* β-glucan composed of β-(1,3)-glucan with β-(1,6)-branching (**Supplementary Figures S1 and S2**) (Lowman et al., 2003b; Sukumaran et al., 2010; Lowman et al., 2014). One parameter that may also contribute to variation in immune responses is the particle size of β-glucans. As revealed by SEM, morphology of *C. albicans* and *C. tropicalis* were quite similar in shape and size whereas the morphology of *C. krusei* was larger and formed different shape (**Figure 1**). Particulate β-glucans differing in size affected the cytokine responses of human monocyte-derived DCs *via* regulation of dectin-1 expression (Elder et al., 2017). The resemblance in morphology and β-glucan structure of *C. albicans* and *C. tropicalis* may contribute to similarity in the immune response observed throughout this study. Presumably, structural diversity and variation in size of *Candida* β-glucans affected their ability to induce DC maturation and cytokine production.

The expression of CD11c was down-modulated by *C. krusei* β-glucans (**Figure 3**), regardless of DC viability (**Supplementary Figure S8**). Low-CD11c expressing *C. krusei* β-glucan-stimulated BMDCs produced high amounts of cytokines (**Figure 5**). Previously, DCs stimulated with several types of PRRs showed CD11c downregulation but these DCs still maintained typical cytokine production and function (Singh-Jasuja et al., 2013; Griffiths et al., 2014). In addition, lower CD11c expression following DC activation was linked with increased cytokine production (Singh-Jasuja et al., 2013). Therefore, down-modulation of CD11c may have indicated certain responses of DCs to stimuli.

DCs play a key role in effector T cell fate decisions, especially *via* cytokine signals (Zhou et al., 2009; Eisenbarth, 2019; Saravia et al., 2019). Our observations from DC:T cell co-culture experiments (**Figure 6**) and *in vivo* immunization of *Candida* β-glucans (**Figure 7**) demonstrated that DC functionality and T cell differentiation were differentially controlled by distinct *Candida* β-glucans (**Figure 6**). Furthermore, T helper cell differentiation was associated with cytokine production by BMDCs mediated by β-glucans. Curdlan-stimulated BMDCs were less capable of driving T cell differentiation (**Figure 6**), which corresponded with the lower ability of curdlan to induce cytokine production in BMDCs (**Figure 10** and **Supplementary Figure S21**). As a consequence of the T cell cytokines (IL-12 and IFN-*γ* for Th1; IL-1β, IL-6, and IL-23 for Th17; IL-10 for regulatory T cells) produced by *Candida* β-glucan-stimulated BMDCs (**Figure 5** and **Supplementary Figure S16**), T cells differentiated into Th1, Th17 and regulatory T cells, which secreted IFN-*γ*, IL-17, and IL-10, respectively (**Figure 6**) (Zhou et al., 2009; Eisenbarth, 2019; Saravia et al., 2019). The increased IL-10 production by T cells activate by *C. krusei*-stimulated BMDCs corresponded with the high IL-10 production by these BMDCs (**Figure 5G** and **Supplementary Figure S16G**); IL-10 is a key cytokine promoting the differentiation of IL-10-secreting regulatory T cells (Hsu et al., 2015; Brockmann et al., 2017; Comi et al., 2018). Although increased CD4^+^ FoxP3^+^ T cells were not detected among T cells co-cultured with *C. krusei* β-glucan-stimulated BMDCs, elevated IL-10 production by T cells probably resulted from the increased function of FoxP3^+^ regulatory T cells and the increased expansion of FoxP3^-^ IL-10-secreting regulatory type 1 T cells (Schmidt et al., 2012). CD4^+^ effector cells (Th1, Th2, and Th17) can also produce IL-10 as a host-protective factor, but the induction of IL-10 in these cells seems to be independent of IL-10 signaling (Jankovic et al., 2010; Ng et al., 2013). In addition to the influence of IL-10 on T cell function and differentiation, substantial expression of co-stimulatory molecules, such as CD80 and CD86 is involved in the induction of IL-10-secreting T cells (Kuipers et al., 2006; Pletinckx et al., 2011). Upregulation of CD86 in BMDCs stimulated with *C. krusei* β-glucan (**Figure 4C** and **Supplementary Figure S15A**) was markedly consistent with the previous reports. Therefore, the enhanced IL-10 production by T cells may have been mediated *via* IL-10 and co-stimulators derived from *C. krusei* β-glucan-stimulated BMDCs. Even though *C. krusei* β-glucan-stimulated BMDCs exhibited high production of Th1 and Th17 cytokines (**Figure 5**), IFN-*γ* and IL-17 production in these T cells was not increased compared with T cells co-cultured with *C. albicans* and *C. tropicalis* β-glucan-stimulated BMDCs (**Figure 6**). These results could be explained by the suppressive effects of IL-10 on IFN-*γ* and IL-17 production as well as Th differentiation (Wang et al., 2016; Neumann et al., 2019).

Since the mice showed the different susceptibility to *Candida* species (Hirayama et al., 2020), it is difficult to compare the immune response among *Candida* species. To determine the immune response to *C. krusei*, we therefore systemically infected mice with low dose and high dose of *C. krusei*. *C. krusei* also mediated IL-17 production in T cells which is similar to a previous report of *C. albicans* infection (LeibundGut-Landmann et al., 2007). However, systemic *C. krusei* infection notably promoted IL-10 production from T cells in a dose-dependent fashion (**Figure 8**). Considering of β-glucans exposed on the outer surface of the cell wall (Wheeler et al., 2008; Gow et al., 2011) and circulating β-glucans in patients with invasive candidiasis (Sims et al., 2012; Giacobbe et al., 2015), β-glucans of *C. krusei* may be one factor that promoted IL-10 production in T cells in murine systemic *C. krusei* infection. While we could observe high IL-10 production in T cells from *C. krusei* infected mice, there was no impact of *C. krusei* in regulatory T cells. The high regulatory T cell number in the control uninfected group may be due to the mechanism of dexamethasone in the induction of regulatory T cell expansion in (Engler et al., 2017). In addition, *C. krusei* infection showed the increased T cell activation (CD4^+^CD25^+^FoxP3^-^) (**Figures 8C, D**), therefore high IL-10 production is possible mediated in a *C. krusei*-specific response.

Recognition of β-glucans by dectin-1 is important for elicitation of host protective immunity required to eradicate *Candida* infection (Taylor et al., 2007). Dectin-1-deficient mice exhibited increased susceptibility to systemic infections caused by various *Candida* species (Chen et al., 2019; Thompson et al., 2019). In addition, dectin-1 dependent immune responses differed among *Candida* species in association with the different levels of β-1,3-glucan exposure on the cell walls (Thompson et al., 2019). Our findings further demonstrated that dectin-1 expression on BMDCs stimulated with different *Candida* β-glucans varied (**Figure 13**), which probably led to differential DC responses (**Figure 9**, **Figure 10**, **Tables 1**, and **Table 2**). It has been well demonstrated that dectin-1 ligation activated Syk tyrosine kinase and led to anti-fungal response in DCs (Whitney et al., 2014). The inhibition of Syk also showed the differential interaction between *Candida* β-glucans and DCs (**Figure 11**, **Supplementary Table 1 and 2**) which supported the results of dectin-1 blockade (**Figure 10**, **Tables 1** and **2**). The differential interaction between dectin-1 with β-glucan was previously demonstrated. The binding affinity of β-glucan to dectin-1 is influenced by polymer chain length and side-chain branching in the β-glucan structure (Adams et al., 2008). The structural conformation of β-glucan also affects its bioactivity and receptor binding efficiency (Sletmoen and Stokke, 2008; Legentil et al., 2015). In addition, the expression level of TLR2 and the cooperative action of dectin-1 and TLR2/TLR4 determine the biological response induced by the dectin-1-β-glucan interaction (Ferwerda et al., 2008; Willcocks et al., 2013; Legentil et al., 2015). Association between dectin-1 and other receptors (galectin-3 and SIGN-R1) may also be involved in dectin-1 signaling mediated by β-glucans (Esteban et al., 2011; Takahara et al., 2011).

We cannot discount the possibility that differential DC responses to distinct *Candida* β-glucans may be influenced by the presence of other β-glucan receptors. Complement receptor 3 (CR3) is a heterodimeric integrin consisting of two membrane proteins, CD11b (αM) and CD18 (β2), and is predominantly expressed on NK cells, DCs, macrophages, monocytes, and neutrophils (Goyal et al., 2018). Recognition of *Candida* β-glucan by CR3 also can activate innate immune cells, leading to *Candida* clearance (Li et al., 2019), and CR3 activation depends on the structure and composition of β-glucan (Legentil et al., 2015). Other receptors, including a lactosylceramide receptor as well as scavenger receptors, are also expressed on innate immune cells and participate in β-glucan recognition. However, little is known regarding how they interact with β-glucan (Zimmerman et al., 1998; Sato et al., 2006; Jozefowski et al., 2012).

A recent study demonstrated that dectin-1 on myeloid cells was required for *C. krusei* killing and induction of subsequent Th1 and Th17 anti-fungal immunity (Chen et al., 2019). In addition, dectin-1 ablation led to high fungal burdens and increased susceptibility to *C. krusei* infection. In contrast with a previous report, we found an immunoregulatory effect of *C. krusei* β-glucan. This discrepancy reflects intra-species diversity in cell wall β-glucans because different strains of *C. krusei* were employed in our study. Carbohydrate analyses of the cell walls of different *C. albicans* strains demonstrated variation in β-glucan structure and composition (Miura et al., 2003; Lowman et al., 2014). In addition, different *C. tropicalis* strains elicited distinct host immune responses (Mesa-Arango et al., 2016). In this study, a single strain of each *Candida* species was employed to investigate immune activation and modulation by inter-species β-glucan variation. Nonetheless, intra-species diversity of cell wall β-glucan should be considered and further investigated.

In this work, we used GM-CSF and IL-4 derived BMDCs as a platform for the *in vitro* study of DCs. The advantage of this culture system is cost-effectiveness and primary BMDCs can produce the results greater than DC cell line. However, BMDCs derived from GM-CSF comprise a heterogeneous population of DCs and macrophages (Helft et al., 2015; McDaniel et al., 2020). The addition of IL-4 in GM-CSF derived DC culture showed ability to limit the number of macrophages (Helft et al., 2015). Therefore, DCs may be the major population in GM-CSF and IL-4 derived BMDCs in our culture system. Nonetheless, the future investigation of DC response to *Candida* β-glucan in other *in vitro* DC culture system such as FLT3L-derived BMDCs will provide more advantage and insight in DC anti-fungal immunity (Brasel et al., 2000).

Our previous study demonstrated the role of *C. krusei* cell wall mannan in DC-mediated Th17 immunity, which could play an important role in anti-*Candida* immunity (Nguyen et al., 2018). Here, we demonstrated the immunomodulatory effects of *C. krusei* cell wall β-glucan, which may either balance or suppress the immune response. Our findings could shed light on the different immune mechanisms responsible for defense against distinct *Candida* species and may be of help in the development of new diagnostic and therapeutic approaches.

## Data Availability Statement

The original contributions presented in the study are included in the article/**Supplementary Material**; further inquiries can be directed to the corresponding author.

## Ethics Statement

All animal procedures were approved by the Chulalongkorn University Institutional Animal Care and Use Committee (IACUC) (Animal protocol 19-33-010).

## Author Contributions

TD performed all dendritic cell experiments and analyzed the data. PT performed the T cell co-culture experiments, *in vivo* experiments, and analyzed the data. PPa and PPo performed the NMR analysis and analyzed the NMR data. LB performed the SEM. WS and AL assisted in the *in vivo* experiment. PK assisted in the *in vivo* data analysis and manuscript writing. PR designed and supervised the research, assisted in the data analyses, processed the data, and wrote and edited manuscript. All authors contributed to the article and approved the submitted version.

## Funding

This work was funded by the National Research Council of Thailand, the Thailand Research Fund (TRF), TSRI fund (CU_FRB640001_01_23_1), the Ratchadapisek Sompoch Endowment Fund, the Chulalongkorn University (Health Cluster 760001-HR), and the Program Management Unit for Human Resources and Institutional Development, Research and Innovation-CU (B16F630071). The Research Unit in Integrative Immuno-Microbial Biochemistry and Bioresponsive Nanomaterials is supported by the Ratchadapisek Sompoch Endowment Fund, Chulalongkorn University.

## Conflict of Interest

The authors declare that the research was conducted in the absence of any commercial or financial relationships that could be construed as a potential conflict of interest.

## References

[B1] Adams E. L. Rice P. J. Graves B. Ensley H. E. Yu H. Brown G. D. (2008). Differential high-affinity interaction of dectin-1 with natural or synthetic glucans is dependent upon primary structure and is influenced by polymer chain length and side-chain branching. J. Pharmacol. Exp. Ther. 325 (1), 115–123. 10.1124/jpet.107.133124 18171906

[B2] Bain J. M. Louw J. Lewis L. E. Okai B. Walls C. A. Ballou E. R. (2014). *Candida albicans* hypha formation and mannan masking of beta-glucan inhibit macrophage phagosome maturation. mBio 5 (6), e01874. 10.1128/mBio.01874-14 25467440 PMC4324242

[B3] Brasel K. De Smedt T. Smith J. L. Maliszewski C. R. (2000). Generation of murine dendritic cells from flt3-ligand-supplemented bone marrow cultures. Blood 96 (9), 3029–3039. 10.1182/blood.V96.9.3029 11049981

[B4] Brockmann L. Gagliani N. Steglich B. Giannou A. D. Kempski J. Pelczar P. (2017). IL-10 Receptor Signaling Is Essential for TR1 Cell Function In Vivo. J. Immunol. 198 (3), 1130–1141. 10.4049/jimmunol.1601045 28003377 PMC5263184

[B5] Brown G. D. Herre J. Williams D. L. Willment J. A. Marshall A. S. Gordon S. (2003). Dectin-1 mediates the biological effects of beta-glucans. J. Exp. Med. 197 (9), 1119–1124. 10.1084/jem.20021890 12719478 PMC2193964

[B6] Camilli G. Tabouret G. Quintin J. (2018). The Complexity of Fungal beta-Glucan in Health and Disease: Effects on the Mononuclear Phagocyte System. Front. Immunol. 9, 673. 10.3389/fimmu.2018.00673 29755450 PMC5932370

[B7] Cardoso L. S. Araujo M. I. Goes A. M. Pacifico L. G. Oliveira R. R. Oliveira S. C. (2007). Polymyxin B as inhibitor of LPS contamination of *Schistosoma mansoni* recombinant proteins in human cytokine analysis. Microb. Cell Fact. 6, 1. 10.1186/1475-2859-6-1 17201926 PMC1766364

[B8] Chen S. M. Zou Z. Qiu X. R. Hou W. T. Zhang Y. Fang W. (2019). The critical role of Dectin-1 in host controlling systemic *Candida krusei* infection. Am. J. Transl. Res. 11 (2), 721–732. 30899374 PMC6413270

[B9] Clement L. T. Tilden A. B. Dunlap N. E. (1985). Analysis of the monocyte Fc receptors and antibody-mediated cellular interactions required for the induction of T cell proliferation by anti-T3 antibodies. J. Immunol. 135 (1), 165–171. 3923099

[B10] Colombo A. L. Junior J. N. A. Guinea J. (2017). Emerging multidrug-resistant *Candida* species. Curr. Opin. Infect. Dis. 30 (6), 528–538. 10.1097/QCO.0000000000000411 29095200

[B11] Comi M. Amodio G. Gregori S. (2018). Interleukin-10-Producing DC-10 Is a Unique Tool to Promote Tolerance Via Antigen-Specific T Regulatory Type 1 Cells. Front. Immunol. 9, 682. 10.3389/fimmu.2018.00682 29686676 PMC5900789

[B12] Davis S. E. Hopke A. Minkin S. C. Jr. Montedonico A. E. Wheeler R. T. Reynolds T. B. (2014). Masking of beta(1-3)-glucan in the cell wall of *Candida albicans* from detection by innate immune cells depends on phosphatidylserine. Infect. Immun. 82 (10), 4405–4413. 10.1128/IAI.01612-14 25114110 PMC4187869

[B13] Eisenbarth S. C. (2019). Dendritic cell subsets in T cell programming: location dictates function. Nat. Rev. Immunol. 19 (2), 89–103. 10.1038/s41577-018-0088-1 30464294 PMC7755085

[B14] Elder M. J. Webster S. J. Chee R. Williams D. L. Hill Gaston J. S. Goodall J. C. (2017). beta-Glucan Size Controls Dectin-1-Mediated Immune Responses in Human Dendritic Cells by Regulating IL-1beta Production. Front. Immunol. 8, 791. 10.3389/fimmu.2017.00791 28736555 PMC5500631

[B15] Engler J. B. Kursawe N. Solano M. E. Patas K. Wehrmann S. Heckmann N. (2017). Glucocorticoid receptor in T cells mediates protection from autoimmunity in pregnancy. Proc. Natl. Acad. Sci. U. S. A. 114 (2), E181–E190. 10.1073/pnas.1617115114 28049829 PMC5240705

[B16] Esteban A. Popp M. W. Vyas V. K. Strijbis K. Ploegh H. L. Fink G. R. (2011). Fungal recognition is mediated by the association of dectin-1 and galectin-3 in macrophages. Proc. Natl. Acad. Sci. U. S. A. 108 (34), 14270–14275. 10.1073/pnas.1111415108 21825168 PMC3161568

[B17] Ferwerda G. Meyer-Wentrup F. Kullberg B. J. Netea M. G. Adema G. J. (2008). Dectin-1 synergizes with TLR2 and TLR4 for cytokine production in human primary monocytes and macrophages. Cell Microbiol. 10 (10), 2058–2066. 10.1111/j.1462-5822.2008.01188.x 18549457

[B18] Gerbst A. G. Grachev A. A. Yashunsky D. V. Tsvetkov Y. E. Shashkov A. S. Nifantiev N. E. (2013). Theoretical and Experimental Conformational Studies of Oligoglucosides Structurally Related to Fragments of Fungal Cell Wall -(13)-D-Glucan. J. Carbohydr. Chem. 32 (3), 205–221. 10.1080/07328303.2013.793347

[B19] Giacobbe D. R. Esteves P. Bruzzi P. Mikulska M. Furfaro E. Mesini A. (2015). Initial serum (1,3)-beta-D-glucan as a predictor of mortality in proven candidaemia: findings from a retrospective study in two teaching hospitals in Italy and Brazil. Clin. Microbiol. Infect. 21 954 (10), e959–e917. 10.1016/j.cmi.2015.06.002 26070961

[B20] Gow N. A. van de Veerdonk F. L. Brown A. J. Netea M. G. (2011). *Candida albicans* morphogenesis and host defence: discriminating invasion from colonization. Nat. Rev. Microbiol. 10 (2), 112–122. 10.1038/nrmicro2711 22158429 PMC3624162

[B21] Goyal S. Castrillon-Betancur J. C. Klaile E. Slevogt H. (2018). The Interaction of Human Pathogenic Fungi With C-Type Lectin Receptors. Front. Immunol. 9, 1261–1645. 10.3389/fimmu.2018.01261 29915598 PMC5994417

[B22] Griffiths K. L. Tan J. K. O’Neill H. C. (2014). Characterization of the effect of LPS on dendritic cell subset discrimination in spleen. J. Cell Mol. Med. 18 (9), 1908–1912. 10.1111/jcmm.12332 24913604 PMC4196665

[B23] Helft J. Bottcher J. Chakravarty P. Zelenay S. Huotari J. Schraml B. U. (2015). GM-CSF Mouse Bone Marrow Cultures Comprise a Heterogeneous Population of CD11c(+)MHCII(+) Macrophages and Dendritic Cells. Immunity 42 (6), 1197–1211. 10.1016/j.immuni.2015.05.018 26084029

[B24] Hirayama T. Miyazaki T. Ito Y. Wakayama M. Shibuya K. Yamashita K. (2020). Virulence assessment of six major pathogenic *Candida* species in the mouse model of invasive candidiasis caused by fungal translocation. Sci. Rep. 10 (1), 3814–3823. 10.1038/s41598-020-60792-y 32123235 PMC7052222

[B25] Hsu P. Santner-Nanan B. Hu M. Skarratt K. Lee C. H. Stormon M. (2015). IL-10 Potentiates Differentiation of Human Induced Regulatory T Cells via STAT3 and Foxo1. J. Immunol. 195 (8), 3665–3674. 10.4049/jimmunol.1402898 26363058

[B26] Inaba K. Swiggard W. J. Steinman R. M. Romani N. Schuler G. Brinster C. (2009). Isolation of dendritic cells. Curr. Protoc. Immunol. Unit 3.7 (Supplement 86), 1–18. 10.1002/0471142735.im0307s86 19653207

[B27] Jacobsen I. D. Brunke S. Seider K. Schwarzmuller T. Firon A. d’Enfert C. (2010). *Candida glabrata* persistence in mice does not depend on host immunosuppression and is unaffected by fungal amino acid auxotrophy. Infect. Immun. 78 (3), 1066–1077. 10.1128/IAI.01244-09 20008535 PMC2825948

[B28] Jamiu A. T. Albertyn J. Sebolai O. M. Pohl C. H. (2020). Update on *Candida krusei*, a potential multidrug-resistant pathogen. Med. Mycol. 10.1093/mmy/myaa031 32400853

[B29] Jankovic D. Kugler D. G. Sher A. (2010). IL-10 production by CD4+ effector T cells: a mechanism for self-regulation. Mucosal Immunol. 3 (3), 239–246. 10.1038/mi.2010.8 20200511 PMC4105209

[B30] Jozefowski S. Yang Z. Marcinkiewicz J. Kobzik L. (2012). Scavenger receptors and beta-glucan receptors participate in the recognition of yeasts by murine macrophages. Inflammation Res. 61 (2), 113–126. 10.1007/s00011-011-0395-5 PMC3265724 22116297

[B31] Kadosh D. Johnson A. D. (2005). Induction of the *Candida albicans* filamentous growth program by relief of transcriptional repression: a genome-wide analysis. Mol. Biol. Cell 16 (6), 2903–2912. 10.1091/mbc.E05-01-0073 15814840 PMC1142434

[B32] Kashem S. W. Igyarto B. Z. Gerami-Nejad M. Kumamoto Y. Mohammed J. A. Jarrett E. (2015). *Candida albicans* morphology and dendritic cell subsets determine T helper cell differentiation. Immunity 42 (2), 356–366. 10.1016/j.immuni.2015.01.008 25680275 PMC4343045

[B33] Katiyar S. K. Edlind T. D. (2001). Identification and expression of multidrug resistance-related ABC transporter genes in *Candida krusei* . Med. Mycol. 39 (1), 109–116. 11270397 10.1080/mmy.39.1.109.116

[B34] Kim S. H. Choi J. K. Cho S. Y. Lee H. J. Park S. H. Choi S. M. (2017). Risk factors and clinical outcomes of breakthrough yeast bloodstream infections in patients with hematological malignancies in the era of newer antifungal agents. Med. Mycol. 56 (2), 197–206. 10.1093/mmy/myx038 PMC5896439 28525644

[B35] Kingeter L. M. Lin X. (2012). C-type lectin receptor-induced NF-kappaB activation in innate immune and inflammatory responses. Cell Mol. Immunol. 9 (2), 105–112. 10.1038/cmi.2011.58 22246129 PMC4002809

[B36] Kono H. Kondo N. Hirabayashi K. Ogata M. Totani K. Ikematsu S. (2017). NMR spectroscopic structural characterization of a water-soluble β-(1→3, 1→6)-glucan from *Aureobasidium pullulans* . Carbohydr. Polym. 174, 876–886. 10.1016/j.carbpol.2017.07.018 28821143

[B37] Kontoyiannis D. P. (2017). Antifungal Resistance: An Emerging Reality and A Global Challenge. J. Infect. Dis. 216 (suppl_3), S431–S435. 10.1093/infdis/jix179 28911044

[B38] Kronen R. Lin C. Hsueh K. Powderly W. Spec A. (2017). Risk Factors and Mortality Associated with *Candida krusei* Bloodstream Infections. Open Forum Infect. Dis. 4 (Suppl 1), S74–S75. 10.1093/ofid/ofx163.008 PMC5808796 29450209

[B39] Kuipers H. Muskens F. Willart M. Hijdra D. van Assema F. B. Coyle A. J. (2006). Contribution of the PD-1 ligands/PD-1 signaling pathway to dendritic cell-mediated CD4+ T cell activation. Eur. J. Immunol. 36 (9), 2472–2482. 10.1002/eji.200635978 16917960

[B40] Legentil L. Paris F. Ballet C. Trouvelot S. Daire X. Vetvicka V. (2015). Molecular Interactions of beta-(1–>3)-Glucans with Their Receptors. Molecules 20 (6), 9745–9766. 10.3390/molecules20069745 26023937 PMC6272582

[B41] LeibundGut-Landmann S. Gross O. Robinson M. J. Osorio F. Slack E. C. Tsoni S. V. (2007). Syk- and CARD9-dependent coupling of innate immunity to the induction of T helper cells that produce interleukin 17. Nat. Immunol. 8 (6), 630–638. 10.1038/ni1460 17450144

[B42] Li Y. Kurlander R. J. (2010). Comparison of anti-CD3 and anti-CD28-coated beads with soluble anti-CD3 for expanding human T cells: differing impact on CD8 T cell phenotype and responsiveness to restimulation. J. Transl. Med. 8, 104. 10.1186/1479-5876-8-104 20977748 PMC2987859

[B43] Li D. Bai C. Zhang Q. Li Z. Shao D. Li X. (2019). beta-1,3-Glucan/CR3/SYK pathway-dependent LC3B-II accumulation enhanced the fungicidal activity in human neutrophils. J. Microbiol. 57 (4), 263–270. 10.1007/s12275-019-8298-1 30721460

[B44] Lortholary O. Renaudat C. Sitbon K. Desnos-Ollivier M. Bretagne S. Dromer F. (2017). The risk and clinical outcome of candidemia depending on underlying malignancy. Intensive Care Med. 43 (5), 652–662. 10.1007/s00134-017-4743-y 28321466 PMC5384959

[B45] Lowman D. W. Ferguson D. A. Williams D. L. (2003a). Structural characterization of (1–>3)-beta-D-glucans isolated from blastospore and hyphal forms of *Candida albicans* . Carbohydr. Res. 338 (14), 1491–1496. 10.1016/s0008-6215(03)00169-1 12829394

[B46] Lowman D. W. Ferguson D. A. Williams D. L. (2003b). Structural characterization of (1→3)-β-d-glucans isolated from blastospore and hyphal forms of Candida albicans. Carbohydr. Res. 338 (14), 1491–1496. 10.1016/S0008-6215(03)00169-1 12829394

[B47] Lowman D. W. Greene R. R. Bearden D. W. Kruppa M. D. Pottier M. Monteiro M. A. (2014). Novel structural features in *Candida albicans* hyphal glucan provide a basis for differential innate immune recognition of hyphae versus yeast. J. Biol. Chem. 289 (6), 3432–3443. 10.1074/jbc.M113.529131 24344127 PMC3916545

[B48] Lu Y. Su C. Liu H. (2014). *Candida albicans* hyphal initiation and elongation. Trends Microbiol. 22 (12), 707–714. 10.1016/j.tim.2014.09.001 25262420 PMC4256103

[B49] Matveev A. L. Krylov V. B. Khlusevich Y. A. Baykov I. K. Yashunsky D. V. Emelyanova L. A. (2019). Novel mouse monoclonal antibodies specifically recognizing beta-(1–>3)-D-glucan antigen. PloS One 14 (4), e0215535. 10.1371/journal.pone.0215535 31022215 PMC6483564

[B50] McDaniel M. M. Kottyan L. C. Singh H. Pasare C. (2020). Suppression of Inflammasome Activation by IRF8 and IRF4 in cDCs Is Critical for T Cell Priming. Cell Rep. 31 (5), 107604–1-14. 10.1016/j.celrep.2020.107604 32375053 PMC7325595

[B51] Mesa-Arango A. C. Rueda C. Roman E. Quintin J. Terron M. C. Luque D. (2016). Cell Wall Changes in Amphotericin B-Resistant Strains from *Candida tropicalis* and Relationship with the Immune Responses Elicited by the Host. Antimicrob. Agents Chemother. 60 (4), 2326–2335. 10.1128/AAC.02681-15 26833156 PMC4808153

[B52] Miura N. N. Adachi Y. Yadomae T. Tamura H. Tanaka S. Ohno N. (2003). Structure and biological activities of beta-glucans from yeast and mycelial forms of *Candida albicans* . Microbiol. Immunol. 47 (3), 173–182. 10.1111/j.1348-0421.2003.tb03382.x 12725286

[B53] Netea M. G. Gijzen K. Coolen N. Verschueren I. Figdor C. Van der Meer J. W. (2004). Human dendritic cells are less potent at killing *Candida albicans* than both monocytes and macrophages. Microbes Infect. 6 (11), 985–989. 10.1016/j.micinf.2004.05.013 15345229

[B54] Netea M. G. Joosten L. A. van der Meer J. W. Kullberg B. J. van de Veerdonk F. L. (2015). Immune defence against *Candida* fungal infections. Nat. Rev. Immunol. 15 (10), 630–642. 10.1038/nri3897 26388329

[B55] Neumann C. Scheffold A. Rutz S. (2019). Functions and regulation of T cell-derived interleukin-10. Semin. Immunol. 44, 101344. 10.1016/j.smim.2019.101344 31727465

[B56] Newman S. L. Holly A. (2001). *Candida albicans* is phagocytosed, killed, and processed for antigen presentation by human dendritic cells. Infect. Immun. 69 (11), 6813–6822. 10.1128/IAI.69.11.6813-6822.2001 11598054 PMC100059

[B57] Ng T. H. Britton G. J. Hill E. V. Verhagen J. Burton B. R. Wraith D. C. (2013). Regulation of adaptive immunity; the role of interleukin-10. Front. Immunol. 4, 129. 10.3389/fimmu.2013.00129 23755052 PMC3668291

[B58] Nguyen T. N. Y. Padungros P. Wongsrisupphakul P. Sa-Ard-Iam N. Mahanonda R. Matangkasombut O. (2018). Cell wall mannan of *Candida krusei* mediates dendritic cell apoptosis and orchestrates Th17 polarization via TLR-2/MyD88-dependent pathway. Sci. Rep. 8 (1), 17123. 10.1038/s41598-018-35101-3 30459422 PMC6244250

[B59] Padungros P. Alberch L. Wei A. (2012). Glycal Assembly by the in Situ Generation of Glycosyl Dithiocarbamates. Organic Lett. 14 (13), 3380–3383. 10.1021/ol301349w PMC3412062 22686424

[B60] Padungros P. Alberch L. Wei A. (2014). Glycosyl Dithiocarbamates: β-Selective Couplings without Auxiliary Groups. J. Organic Chem. 79 (6), 2611–2624. 10.1021/jo500032k PMC3985838 24548247

[B61] Palma A. S. Feizi T. Zhang Y. Stoll M. S. Lawson A. M. Diaz-Rodriguez E. (2006). Ligands for the beta-glucan receptor, Dectin-1, assigned using “designer” microarrays of oligosaccharide probes (neoglycolipids) generated from glucan polysaccharides. J. Biol. Chem. 281 (9), 5771–5779. 10.1074/jbc.M511461200 16371356

[B62] Pletinckx K. Dohler A. Pavlovic V. Lutz M. B. (2011). Role of dendritic cell maturity/costimulation for generation, homeostasis, and suppressive activity of regulatory T cells. Front. Immunol. 2, 39. 10.3389/fimmu.2011.00039 22566829 PMC3342346

[B63] Saravia J. Chapman N. M. Chi H. (2019). Helper T cell differentiation. Cell Mol. Immunol. 16 (7), 634–643. 10.1038/s41423-019-0220-6 30867582 PMC6804569

[B64] Sato T. Iwabuchi K. Nagaoka I. Adachi Y. Ohno N. Tamura H. (2006). Induction of human neutrophil chemotaxis by *Candida albicans*-derived beta-1,6-long glycoside side-chain-branched beta-glucan. J. Leukoc. Biol. 80 (1), 204–211. 10.1189/jlb.0106069 16670126

[B65] Schmidt A. Oberle N. Krammer P. H. (2012). Molecular mechanisms of Treg-mediated T cell suppression. Front. Immunol. 3, 51. 10.3389/fimmu.2012.00051 22566933 PMC3341960

[B66] Serrano I. Luque A. Aran J. M. (2018). Exploring the Immunomodulatory Moonlighting Activities of Acute Phase Proteins for Tolerogenic Dendritic Cell Generation. Front. Immunol. 9, 892. 10.3389/fimmu.2018.00892 29760704 PMC5936965

[B67] Sims C. R. Jaijakul S. Mohr J. Rodriguez J. Finkelman M. Ostrosky-Zeichner L. (2012). Correlation of clinical outcomes with beta-glucan levels in patients with invasive candidiasis. J. Clin. Microbiol. 50 (6), 2104–2106. 10.1128/JCM.00773-12 22461680 PMC3372136

[B68] Singh-Jasuja H. Thiolat A. Ribon M. Boissier M. C. Bessis N. Rammensee H. G. (2013). The mouse dendritic cell marker CD11c is down-regulated upon cell activation through Toll-like receptor triggering. Immunobiology 218 (1), 28–39. 10.1016/j.imbio.2012.01.021 22445076

[B69] Skrzypek F. Cenci E. Pietrella D. Rachini A. Bistoni F. Vecchiarelli A. (2009). Dectin-1 is required for human dendritic cells to initiate immune response to *Candida albicans* through Syk activation. Microbes Infect. 11 (6-7), 661–670. 10.1016/j.micinf.2009.03.010 19358895

[B70] Sletmoen M. Stokke B. T. (2008). Higher order structure of (1,3)-beta-D-glucans and its influence on their biological activities and complexation abilities. Biopolymers 89 (4), 310–321. 10.1002/bip.20920 18186085

[B71] Spolski R. Li P. Leonard W. J. (2018). Biology and regulation of IL-2: from molecular mechanisms to human therapy. Nat. Rev. Immunol. 18 (10), 648–659. 10.1038/s41577-018-0046-y 30089912

[B72] Sukumaran V. Lowman D. W. Sajeevan T. P. Philip R. (2010). Marine yeast glucans confer better protection than that of baker’s yeast in *Penaeus monodon* against white spot syndrome virus infection. Aquacult. Res. 41 (12), 1799–1805. 10.1111/j.1365-2109.2010.02520.x

[B73] Suzuki T. Omi K. Ito T. Takata T. Kikutsugi H. Fujimura T. (2006). Ethanol-induced pseudohyphal transition in the cells of *Candida tropicalis*: participation of phosphoinositide signal transduction. FEMS Yeast Res. 6 (2), 177–185. 10.1111/j.1574-1364.2005.00020.x 16487341

[B74] Takahara K. Tokieda S. Nagaoka K. Takeda T. Kimura Y. Inaba K. (2011). C-type lectin SIGNR1 enhances cellular oxidative burst response against *C. albicans* in cooperation with Dectin-1. Eur. J. Immunol. 41 (5), 1435–1444. 10.1002/eji.200940188 21400494

[B75] Takano T. Motozono C. Imai T. Sonoda K. H. Nakanishi Y. Yamasaki S. (2017). Dectin-1 intracellular domain determines species-specific ligand spectrum by modulating receptor sensitivity. J. Biol. Chem. 292 (41), 16933–16941. 10.1074/jbc.M117.800847 28848046 PMC5641891

[B76] Takeuchi O. Akira S. (2010). Pattern recognition receptors and inflammation. Cell 140 (6), 805–820. 10.1016/j.cell.2010.01.022 20303872

[B77] Taylor P. R. Tsoni S. V. Willment J. A. Dennehy K. M. Rosas M. Findon H. (2007). Dectin-1 is required for beta-glucan recognition and control of fungal infection. Nat. Immunol. 8 (1), 31–38. 10.1038/ni1408 17159984 PMC1888731

[B78] Thompson D. S. Carlisle P. L. Kadosh D. (2011). Coevolution of morphology and virulence in *Candida* species. Eukaryot. Cell 10 (9), 1173–1182. 10.1128/EC.05085-11 21764907 PMC3187052

[B79] Thompson A. Griffiths J. S. Walker L. da Fonseca D. M. Lee K. K. Taylor P. R. (2019). Dependence on Dectin-1 Varies With Multiple *Candida Species* . Front. Microbiol. 10, 1800. 10.3389/fmicb.2019.01800 31447813 PMC6691182

[B80] Wang S. Gao X. Shen G. Wang W. Li J. Zhao J. (2016). Interleukin-10 deficiency impairs regulatory T cell-derived neuropilin-1 functions and promotes Th1 and Th17 immunity. Sci. Rep. 6, 24249. 10.1038/srep24249 27075020 PMC4831052

[B81] Wheeler R. T. Kombe D. Agarwala S. D. Fink G. R. (2008). Dynamic, morphotype-specific *Candida albicans* beta-glucan exposure during infection and drug treatment. PloS Pathog. 4 (12), e1000227. 10.1371/journal.ppat.1000227 19057660 PMC2587227

[B82] Whitney P. G. Bar E. Osorio F. Rogers N. C. Schraml B. U. Deddouche S. (2014). Syk signaling in dendritic cells orchestrates innate resistance to systemic fungal infection. PloS Pathog. 10 (7), e1004276. 10.1371/journal.ppat.1004276 25033445 PMC4102599

[B83] Willcocks S. Offord V. Seyfert H. M. Coffey T. J. Werling D. (2013). Species-specific PAMP recognition by TLR2 and evidence for species-restricted interaction with Dectin-1. J. Leukoc. Biol. 94 (3), 449–458. 10.1189/jlb.0812390 23787127

[B84] Wuthrich M. Deepe G. S. Jr. Klein B. (2012). Adaptive immunity to fungi. Annu. Rev. Immunol. 30, 115–148. 10.1146/annurev-immunol-020711-074958 22224780 PMC3584681

[B85] Yamanaka D. Takatsu K. Kimura M. Swamydas M. Ohnishi H. Umeyama T. (2020). Development of a novel beta-1,6-glucan-specific detection system using functionally-modified recombinant endo-beta-1,6-glucanase. J. Biol. Chem. 295 (16), 5362–5376. 10.1074/jbc.RA119.011851 PMC7170528 32132174

[B86] Zhou L. Chong M. M. Littman D. R. (2009). Plasticity of CD4+ T cell lineage differentiation. Immunity 30 (5), 646–655. 10.1016/j.immuni.2009.05.001 19464987

[B87] Zimmerman J. W. Lindermuth J. Fish P. A. Palace G. P. Stevenson T. T. DeMong D. E. (1998). A novel carbohydrate-glycosphingolipid interaction between a beta-(1-3)-glucan immunomodulator, PGG-glucan, and lactosylceramide of human leukocytes. J. Biol. Chem. 273 (34), 22014–22020. 10.1074/jbc.273.34.22014 9705343

