# Supplementary material for: Interaction Between Dendritic Cells and Candida krusei β-Glucan Partially Depends on Dectin-1 and It Promotes High IL-10 Production by T Cells
Source: Front Cell Infect Microbiol. 2021 Jan 22;10:566661. doi: 10.3389/fcimb.2020.566661 (PMC7862133; doi:10.3389/fcimb.2020.566661)
Supplement: Supplementary file 1 [file DataSheet_1.docx]

**Supplementary Information**

**Interaction between dendritic cells and *Candida krusei* β-glucan partially depends on dectin-1 and it promotes high IL-10 production by T cells**

Truc Thi Huong Dinh^1,2^, Phawida Tummamunkong^2^, Panuwat Padungros^3^,

Pranpariya Ponpakdee^3^, Lawan Boonprakong^4^, Wilasinee Saisorn^5^,

Asada Leelahavanichkul^5^, Patipark Kueanjinda^6^, Patcharee Ritprajak^2,7*^

^1^Medical Microbiology Interdisciplinary Program, Graduate School, Chulalongkorn University, Bangkok, Thailand

^2^Research Unit in Integrative Immuno-Microbial Biochemistry and Bioresponsive Nanomaterials, Department of Microbiology, Faculty of Dentistry, Chulalongkorn University, Bangkok, Thailand

^3^Green Chemistry for Fine Chemical Productions STAR, Department of Chemistry, Faculty of Science, Chulalongkorn University, Bangkok, Thailand

^4^Oral Biology Research Center, Faculty of Dentistry, Chulalongkorn University, Bangkok, Thailand

^5^Translational Research in Inflammation and Immunology Research Unit (TRIRU), Department of Microbiology, Faculty of Medicine, Chulalongkorn University, Bangkok, Thailand

^6^Department of Microbiology, Faculty of Medicine, Chulalongkorn University

^7^Department of Microbiology, Faculty of Dentistry, Chulalongkorn University, Bangkok, Thailand

*** Correspondence:**

**Corresponding Author**

Patcharee Ritprajak, DDS, PhD, Assistant Professor

Department of Microbiology, Faculty of Dentistry, Chulalongkorn University

34 Henri-Dunant Road, Pathumwan, Bangkok, 10330, Thailand

Tel & Fax: +662-218-8680

E-mail address: [Patcharee.R@chulalac.th](mailto:Patcharee.R@chulalac.th)

**Keywords:** *Candida krusei*, β-glucan, dendritic cells, T cells, dectin-1, immune modulation

**Running Title:** Dendritic cell-*Candida krusei* β-glucan interaction

**Supplementary materials and methods**

**Structure analysis by NMR spectroscopy**

Chemical structure characterization was conducted by nuclear magnetic resonance (NMR) spectrometer on a JEOL JNM-ECZ500R/S1 spectrometer operating at 500 MHz for ^1^H NMR and 126 MHz for ^13^C NMR. For *C. albicans* β-glucan and *C. tropicalis* β-glucan analysis, 50 mg of β-glucan was dissolved in 1.57 mL of DMSO-d_6_ and 0.08 mL of TFA-d_1_. For *C. krusei* β-glucan analysis, 50 mg of β-glucan was dissolved in 2.07 mL of DMSO-d_6_ and 0.11 mL TFA-d_1_. Then approximately 0.5 mL of each solution was withdrawn to perform the NMR experiment at room temperature. NMR chemical shifts were referenced to the residual DMSO-d_6_ proton at δ 2.54 ppm and carbon at δ 40.45 ppm.

**Polymyxin B treatment**

*Candida* β-glucans were treated with Polymyxin B (GIBCO) at the concentration of 25 units/ml and 50 units/ml at room temperature in dark for 2 h with rotation. The glucans were then washed twice with culture media. BMDCs were stimulated with polymyxin B-treated β glucans (25 μg/ml). The culture supernatants were collected for IL-6 and TNF-α measurement by ELISA. At 24 and 48 h after stimulation, the cells were harvested and stained for DC maturation markers CD40, CD80, CD86 and MHC class II. The stained cells were assessed by flow cytometry.

**Cell viability assessment**

BMDCs (1×10^5^ cells per 200 µL) were cultured in 96-well plates and stimulated with various concentrations of *Candida* β-glucans. At 24 h and 48 h post-stimulation, 20 µL of 12 mM 3-(4,5-dimethylthiazol-2-yl)-2,5-diphenyltetrazolium bromide (MTT; Invitrogen, Carlsbad, CA, USA) were added to each well and the cells were then incubated at 37^o^C in the dark under a humidified atmosphere containing 5% CO_2_ for 1 h. The culture media were removed and the cells were gently washed twice with Dulbecco’s phosphate-buffered saline (GIBCO). Subsequently, the purple formazan crystals incorporated in viable cells were solubilized in dimethyl sulfoxide (AMRESCO, Cleveland, OH, USA). The absorbance at 570 nm was measured using a microplate reader (EPCHO2, BioTek, Winooski, VT, USA). The percent cell viability was calculated by normalization to the negative control.

**Supplementary Figure S1**

**^1^H NMR spectrum of *C. albicans* β-glucan (500 MHz, DMSO-d6 + TFA-d)**

*C. albicans* β-glucan 50 mg was dissolved in DMSO-d_6_ (1.57 mL) and TFA-d (0.08 mL) then 0.5 mL of the solution was withdrawn to perform the ^1^H NMR experiment with 500 MHz NMR spectrometer at room temperature.

**Supplementary Figure S2**

**^1^H NMR spectrum of *C. tropicalis* β-glucan (500 MHz, DMSO-d6 + TFA-d)**

*C. tropicalis* β-glucan 50 mg was dissolved in DMSO-d_6_ (1.57 mL) and TFA-d (0.08 mL) then 0.5 mL of the solution was withdrawn to perform the ^1^H NMR experiment with 500 MHz NMR spectrometer at room temperature.

**Supplementary Figure S3**

**^1^H NMR spectrum of *C. krusei* β-glucan (500 MHz, DMSO-d6 + TFA-d)**

*C. krusei* β-glucan 50 mg was dissolved in DMSO-d_6_ (2.07 mL) and TFA-d (0.11 mL) then 0.5 mL of the solution was withdrawn to perform the ^1^H NMR experiment with 500 MHz NMR spectrometer at room temperature.

**Supplementary Figure S4**

**^13^C NMR spectrum of *C. krusei* β-glucan (126 MHz, DMSO-d6 + TFA-d)**

*C. krusei* β-glucan 50 mg was dissolved in DMSO-d_6_ (2.07 mL) and TFA-d (0.11 mL) then 0.5 mL of the solution was withdrawn to perform the ^13^C NMR experiment with 126 MHz NMR spectrometer at room temperature.

**Supplementary Figure S5**

**COSY NMR spectrum of *C. krusei* β-glucan (500 MHz, DMSO-d6 + TFA-d)**

*C. krusei* β-glucan 50 mg was dissolved in DMSO-d_6_ (2.07 mL) and TFA-d (0.11 mL) then 0.5 mL of the solution was withdrawn to perform the COSY NMR experiment with 500 MHz NMR spectrometer at room temperature.


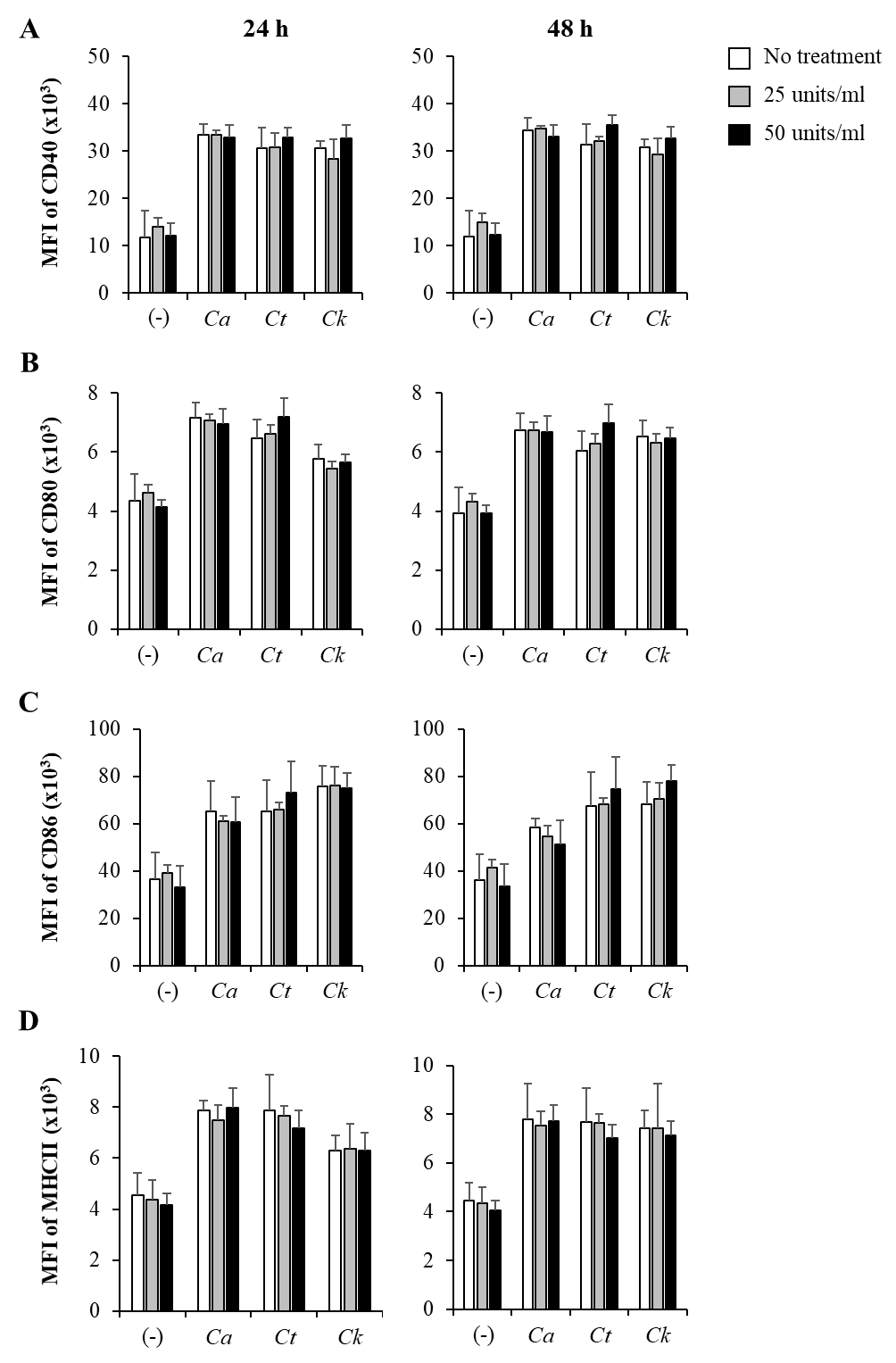


**Supplementary Figure S6**

**DC maturation after stimulation with Candida β-glucans pre-treated with endotoxin removal polymyxin B**

*Candida* β-glucans were pre-treated with 25 and 50 units/ml polymyxin B for 2 h at room temperature with rotation. The glucans were washed twice with PBS. BMDCs were stimulated with polymyxin B-treated β-glucans (25 μg/ml) for 24 h and 48 h. DC maturation markers were assessed by flow cytometer.


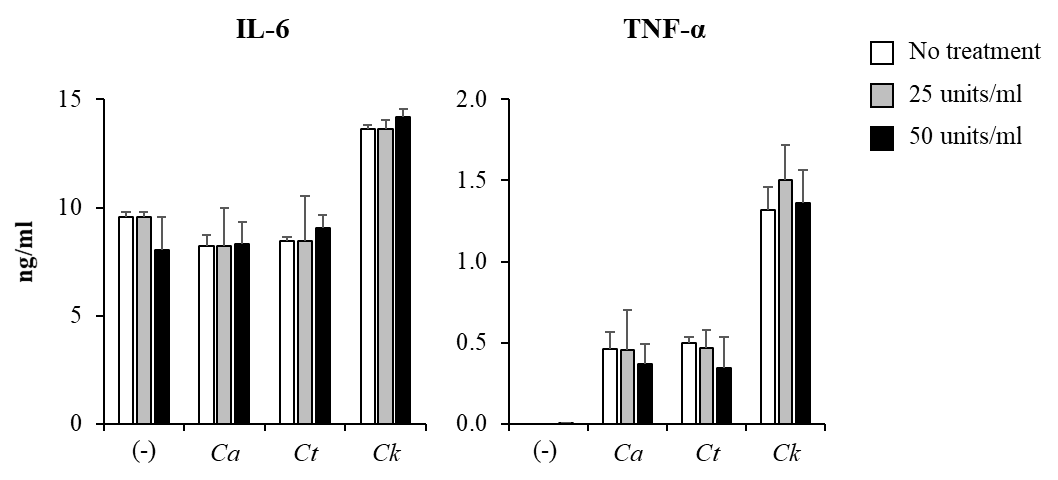


**Supplementary Figure S7**

**Cytokine expression after stimulation with Candida β-glucans pre-treated with endotoxin removal polymyxin B**

*Candida* β-glucans were pre-treated with 25 and 50 units/ml polymyxin B for 2 h at room temperature with rotation. The glucans were washed twice with PBS. BMDCs were stimulated with polymyxin B-treated β-glucans (25 μg/ml) for 24 h and 48 h. Cytokine expression levels were assessed by ELISA.


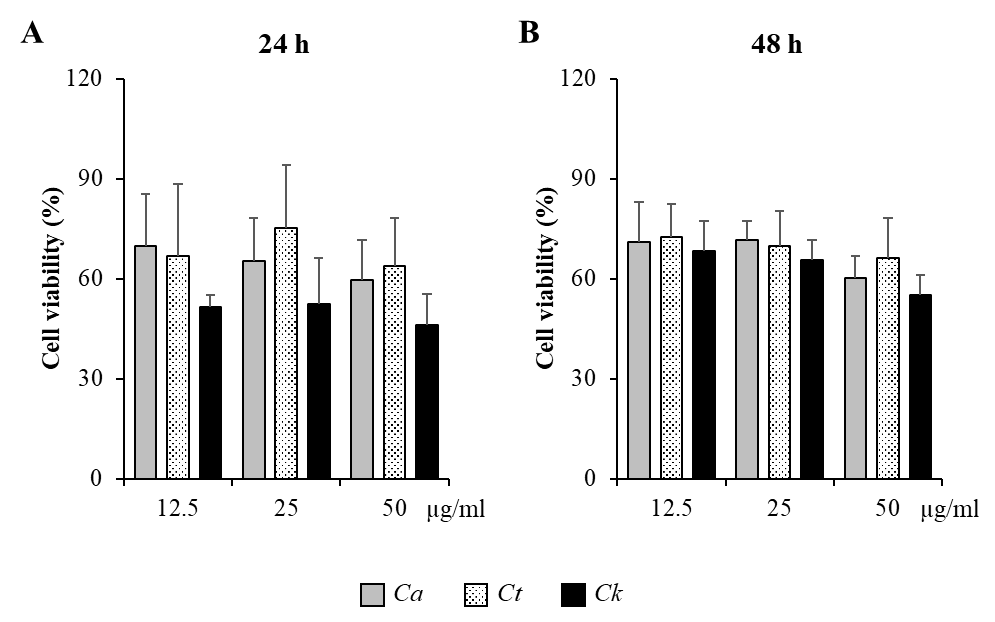


**Supplementary Figure S8**

**Effects of *Candida* β-glucans** **on DC viability**

BMDCs were stimulated with 12.5, 25 and 50 µg/ml of β-glucans isolated from *C. albicans*, *C. tropicalis*, and *C. krusei* for **(A)** 24 h and **(B)** 48 h. DC viability was evaluated using a MTT assay. The percent cell viability was calculated by normalization to the negative control. *n* = 5; data are representative of two independent experiments. *Ca*, *C. albicans*; *Ct*, *C. tropicalis*; *Ck*, *C. krusei*.


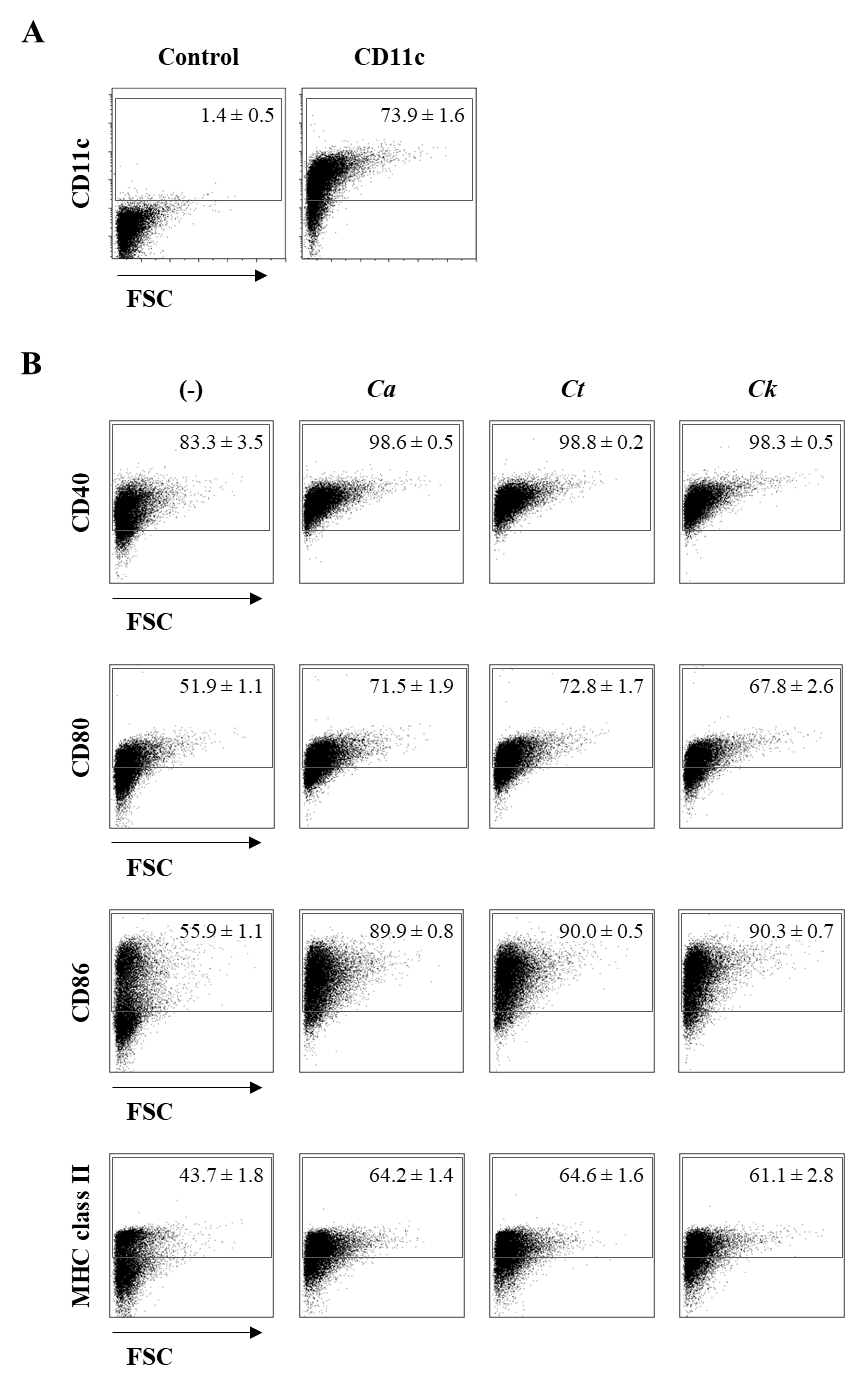


**Supplementary Figure S9**

**Dot plot analyses of CD11c and DC maturation markers at 24 h**

BMDCs were stimulated with *Candida* β-glucans for 24 h. DC proportion and mature DC populations were analyzed by a dot pot analysis. **(A)** DCs were identified by gating on CD11c^+^ cells. The left panel is isotype control staining, and the right panel is CD11c staining. **(B)** Dot plot analyses of the mature DC populations in CD11c^+^ cells. The plots are representative of the data of BMDCs stimulated with 25 μg/ml of *Candida* β-glucans. The values indicated the percentages of the positive cells (mean ± S.D.).


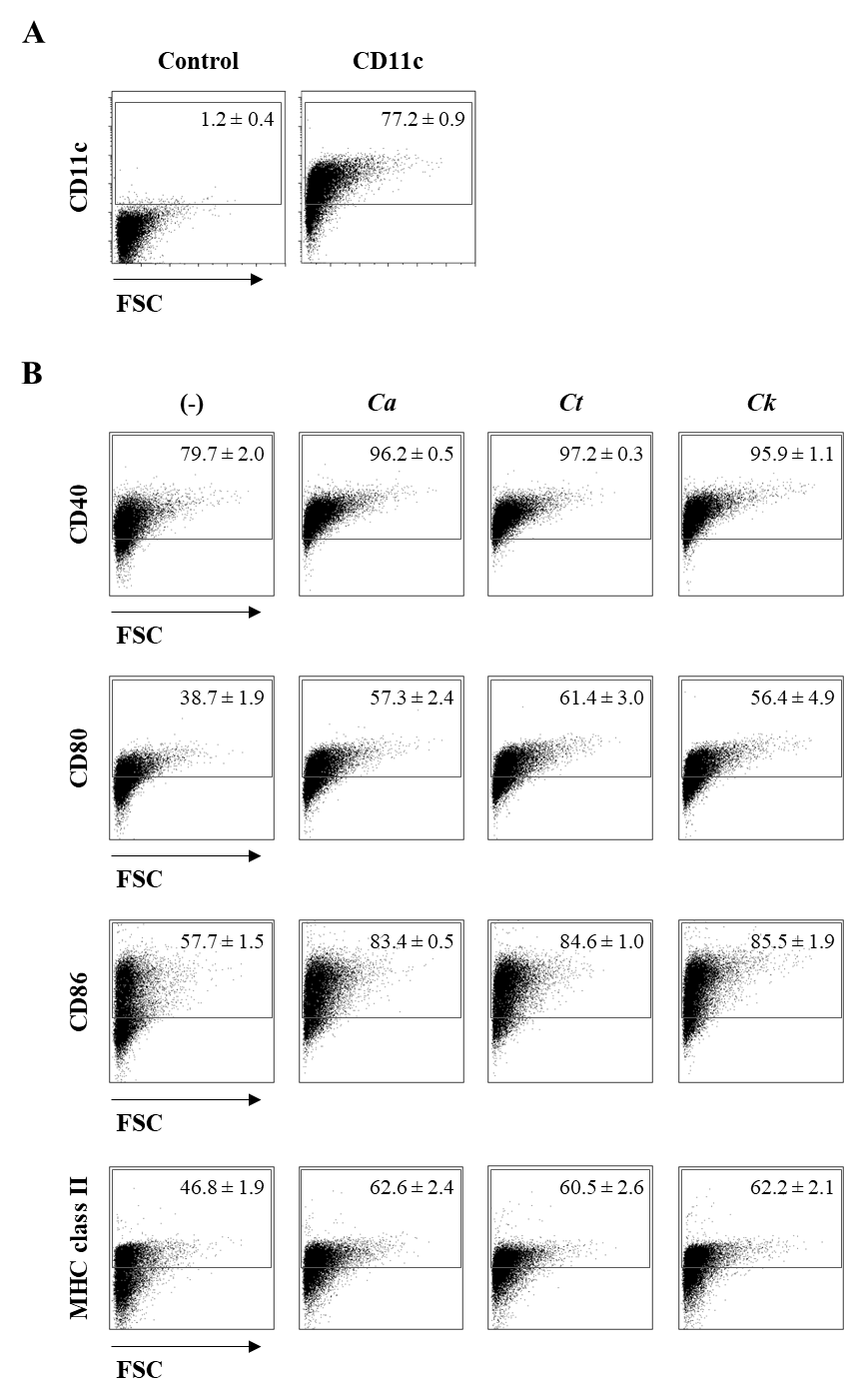


**Supplementary Figure S10**

**Dot plot analyses of CD11c and DC maturation markers at 48 h**

BMDCs were stimulated with *Candida* β-glucans for 48 h. DC proportion and mature DC populations were analyzed by a dot pot analysis. **(A)** DCs were identified by gating on CD11c^+^ cells. The left panel is isotype control staining, and the right panel is CD11c staining. **(B)** Dot plot analyses of the mature DC populations in CD11c^+^ cells. The plots are representative of the data of BMDCs stimulated with 25 μg/ml of *Candida* β-glucans. The values indicated the percentages of the positive cells (mean ± S.D.). (-), unstimulated BMDCs; *Ca*, *C. albicans*; *Ct*, *C. tropicalis*; *Ck*, *C. krusei*.


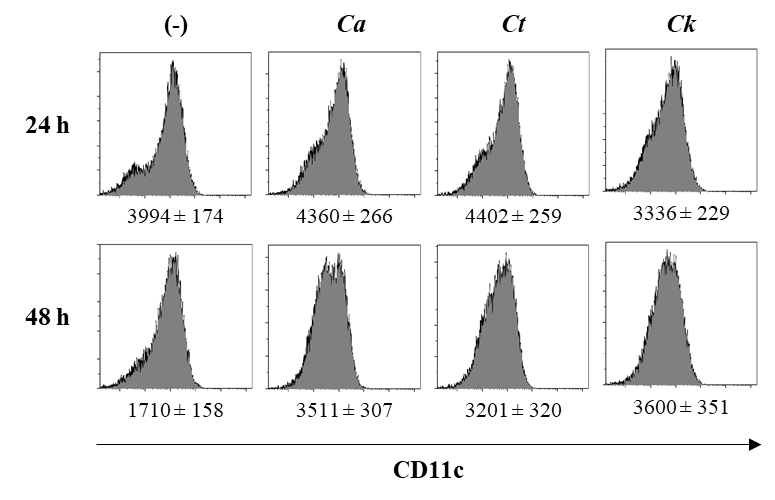


**Supplementary Figure S11**

**Histogram analysis of CD11c expression**

BMDCs were stimulated with *Candida* β-glucans for 24 and 48 h, and CD11c expression was then assessed by a histogram analysis. The plots are representative of the data of BMDCs stimulated with 25 μg/ml of *Candida* β-glucans. The values indicated the MFI of CD11c (mean ± S.D.). (-), unstimulated BMDCs; *Ca*, *C. albicans*; *Ct*, *C. tropicalis*; *Ck*, *C. krusei*.


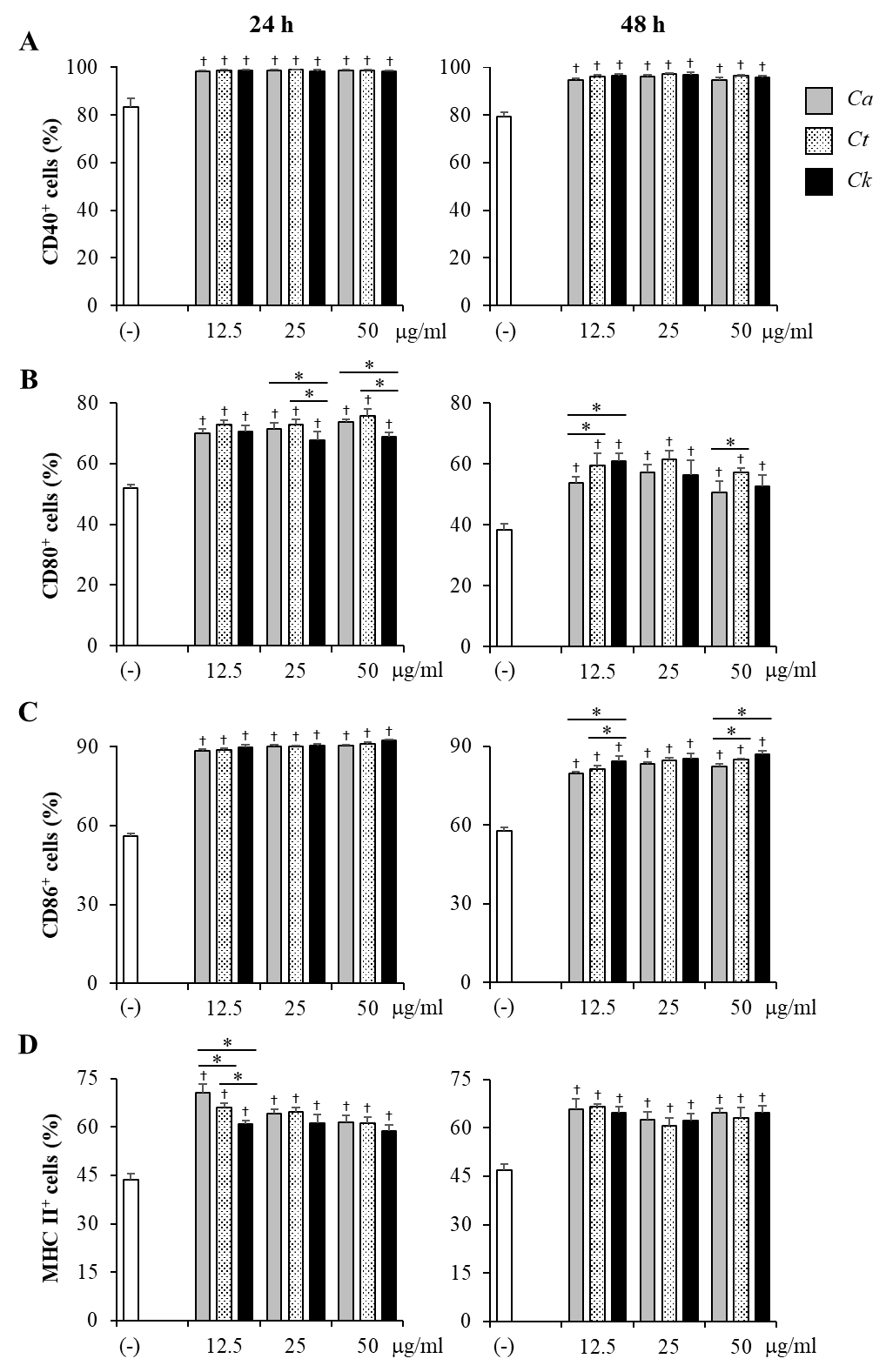


**Supplementary Figure S12**

**Effects of *Candida* β-glucans** **on the proportions of mature DCs**

BMDCs were stimulated with 12.5, 25 and 50 µg/mL of β-glucans isolated from *C. albicans*, *C. tropicalis*, and *C. krusei* for 24 h and 48 h. Cells were analyzed by flow cytometry. The percentages of **(A)** CD40^+^ cells, **(B)** CD80^+^ cells, **(C)** CD86^+^ cells, and **(D)** MHC class II^+^ cells in CD11c^+^ population were assessed using dot plot analyses. *n* = 5; data are representative of two independent experiments. ✝ *p*<0.05 compared with unstimulated BMDCs, * *p*<0.05. (-), unstimulated BMDCs; *Ca*, *C. albicans*; *Ct*, *C. tropicalis*; *Ck*, *C. krusei*.


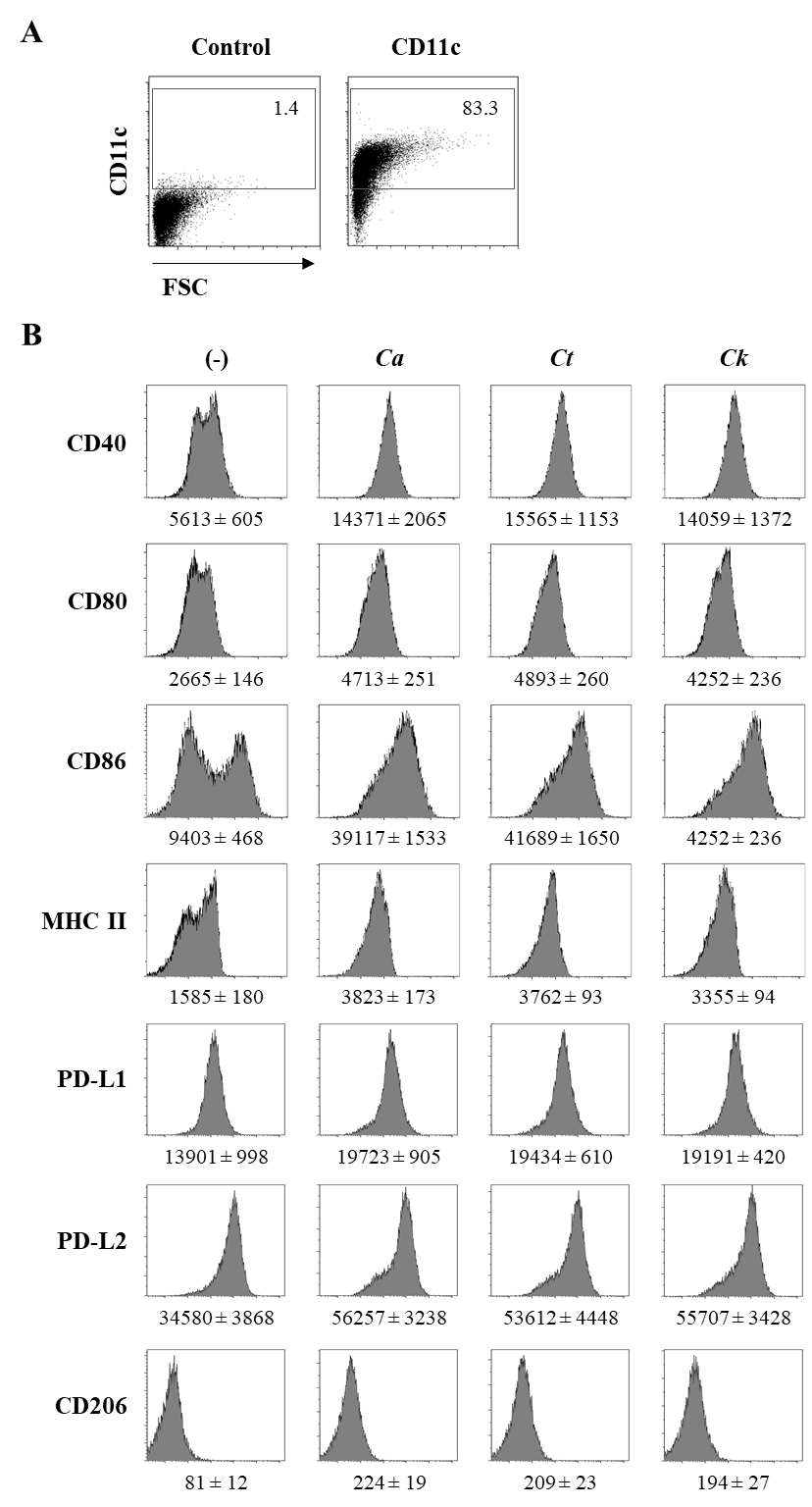


**Supplementary Figure S13**

**Flow cytometric analyses of DC maturation at 24 h**

BMDCs were stimulated with *Candida* β-glucans for 24 h, and the expression of DC maturation markers were determined by flow cytometric analyses. DCs were first identified by gating on CD11c as in **(A)**. Then, the expression of the DC maturation markers was analyzed using histogram analyses. The plots and histograms The plots are representative of the data of BMDCs stimulated with 25 μg/ml of *Candida* β-glucans. The values in **(A)** indicated the percentages of the positive cells. The values in **(B)** indicated the MFI (mean ± S.D.) of CD40, CD80, CD86, MHC class II, PD-L1, PD-L2 and CD206. (-), unstimulated BMDCs; *Ca*, *C. albicans*; *Ct*, *C. tropicalis*; *Ck*, *C. krusei*.


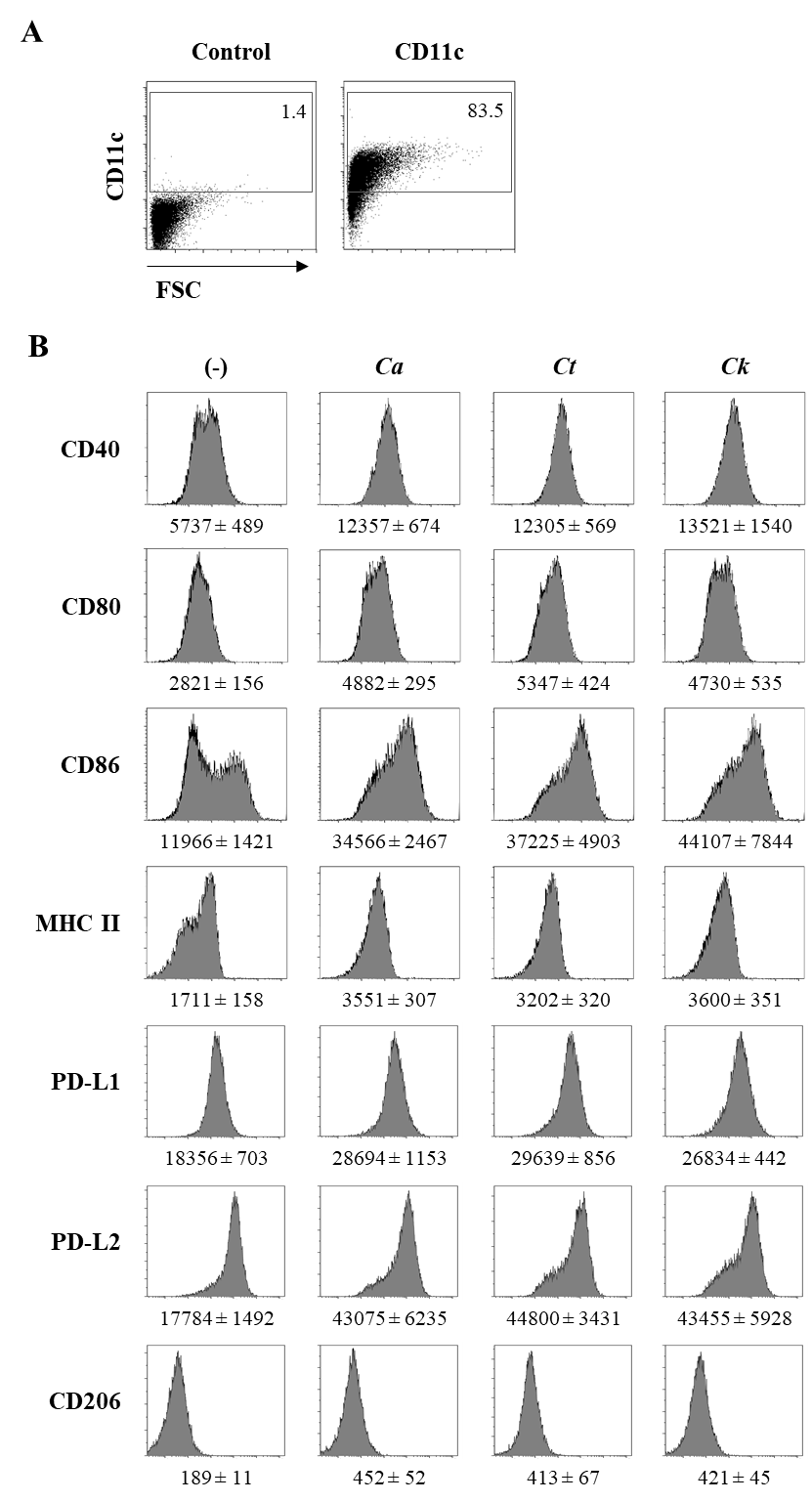


**Supplementary Figure S14**

**Flow cytometric analyses of DC maturation at 48 h**

BMDCs were stimulated with *Candida* β-glucans for 48 h, and the expression of DC maturation markers were determined by flow cytometric analyses. DCs were first identified by gating on CD11c as in **(A)**. Then, the expression of the DC maturation markers was analyzed using histogram analyses. The plots and histograms. The plots are representative of the data of BMDCs stimulated with 25 μg/ml of *Candida* β-glucans. The values in **(A)** indicated the percentages of the positive cells. The values in **(B)** indicated the MFI (mean ± S.D.) of CD40, CD80, CD86, MHC class II, PD-L1, PD-L2 and CD206. (-), unstimulated BMDCs; *Ca*, *C. albicans*; *Ct*, *C. tropicalis*; *Ck*, *C. krusei*.


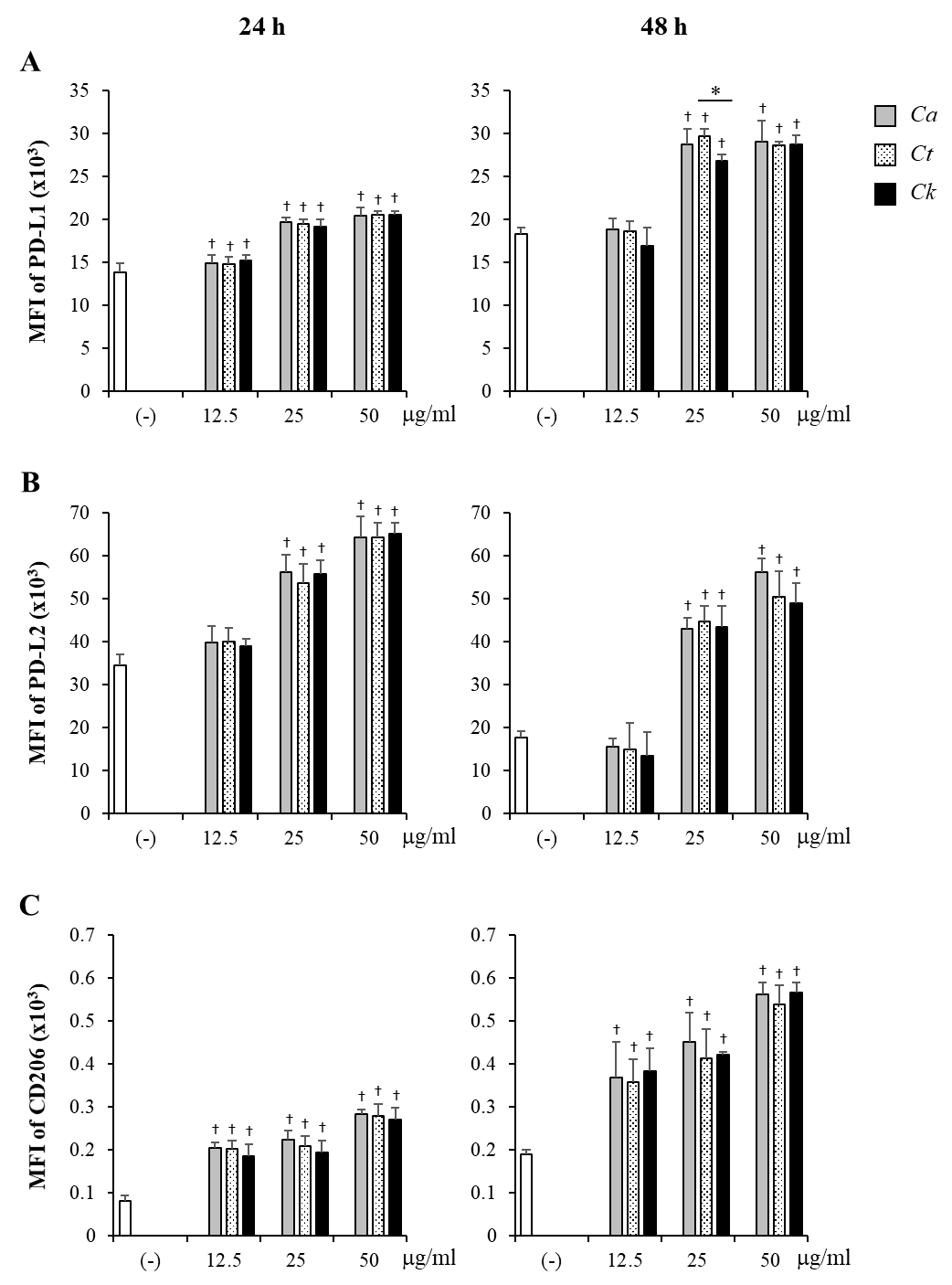


**Supplementary Figure S15**

**Expression levels of surface protein PD-L1, PD-L2, CD206 on BMDCs stimulated with *Candida* β-glucans**

BMDCs were stimulated with 12.5, 25 and 50 µg/mL of β-glucans isolated from *C. albicans*, *C. tropicalis*, and *C. krusei* for 24 h and 48 h, and the geometric mean fluorescence intensity (MFI) of tolerogenic markers **(A)** PD-L1, **(B)** PD-L2, and **(C)** CD206 on CD11c^+^ cells were determined using histogram analyses. *n* = 5; data are representative of two independent experiments. ✝ *p*<0.05 compared with unstimulated BMDCs, * *p*<0.05. (-), unstimulated BMDCs; *Ca*, *C. albicans*; *Ct*, *C. tropicalis*; *Ck*, *C. krusei*.


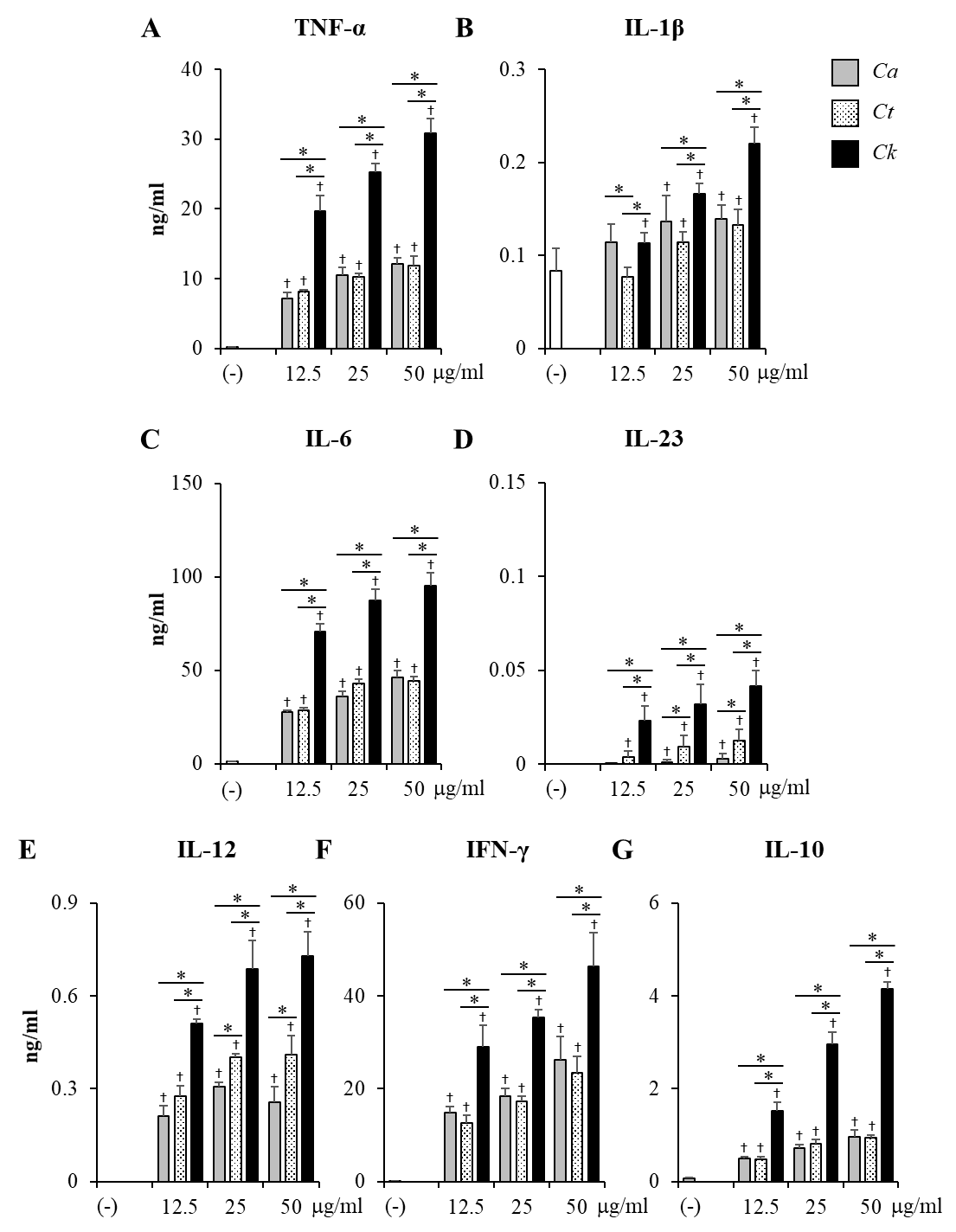


**Supplementary Figure S16**

**Cytokine profiles of *Candida* β-glucan-stimulated BMDCs at 48 h**

BMDCs were stimulated with 12.5, 25 and 50 µg/ml of β-glucans isolated from *C. albicans*, *C. tropicalis*, and *C. krusei* for 48 h. Subsequently, the culture supernatants were collected, and levels of **(A)** TNF-α, **(B)** IL-1β, **(C)** IL-6, **(D)** IL-23, **(E)** IL-12, **(F)** IFN-γ, and **(G)** IL-10 were quantitated by ELISA. *n* = 5; Data are representatives of two independent experiments. ✝ *p*<0.05 compared with unstimulated BMDCs, * *p*<0.05. (-), unstimulated BMDCs; *Ca*, *C. albicans*; *Ct*, *C. tropicalis*; *Ck*, *C. krusei*.


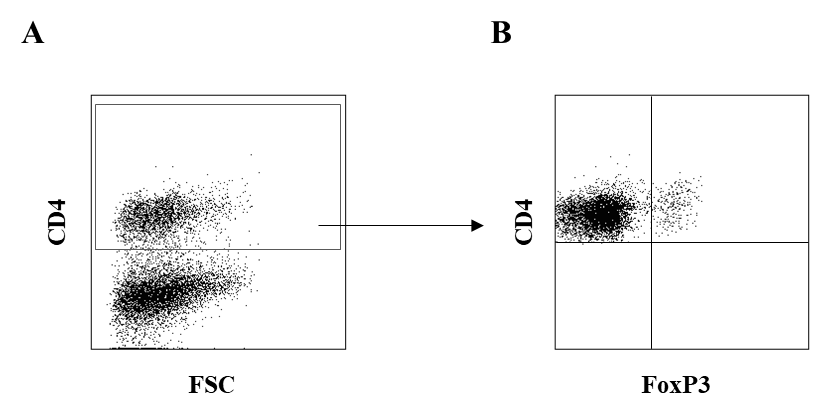


**Supplementary Figure S17**

**Representative of dot plot analyses of CD4^+^ T cells and CD4^+^FoxP3^+^ T cells**

**(A)** CD4 T cells were first identified by gating on CD4^+^ cells. **(B)** FoxP3^+^ regulatory T cells in CD4^+^ T cell population were next identified by gating on CD4^+^ FoxP3^+^ cells.


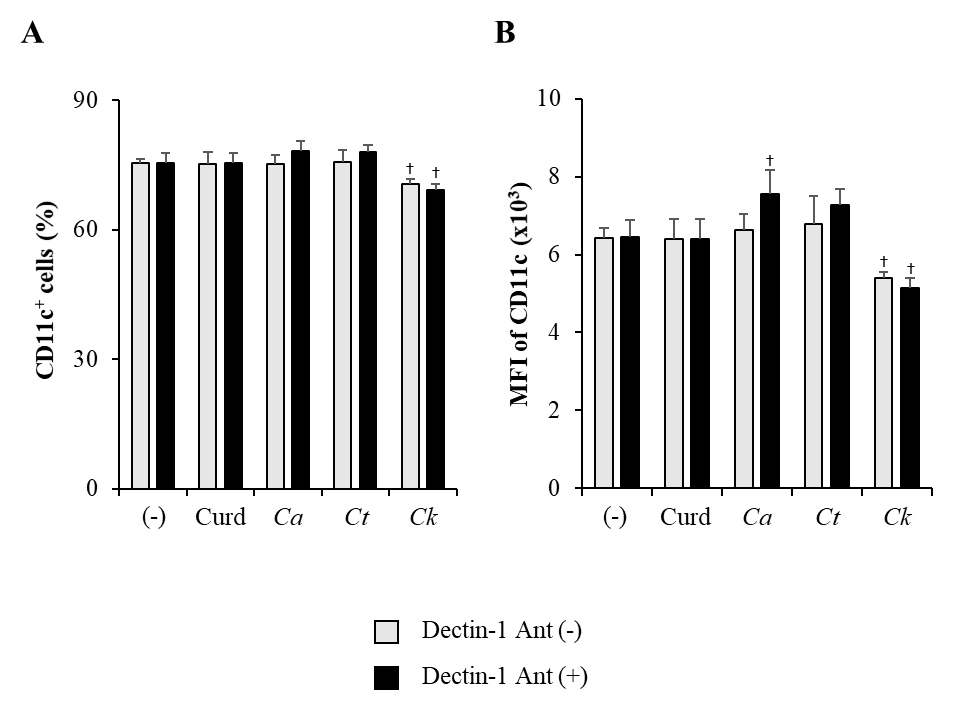


**Figure S18**

**Impact of dectin-1 blockade on DC proportion and CD11c expression**

BMDCs were pre-treated with 25 µg/mL of dectin-1 antagonist for 2 h, then cells were stimulated with 25 µg/mL of curdlan and β-glucans isolated from *C. albicans*, *C. tropicalis*, and *C. krusei* for 24 h. **(A)** The percentages of CD11c^+^ cells were assessed using a dot pot analysis. **(B)** The geometric mean fluorescence intensity (MFI) of CD11c was determined using a histogram analysis. *n* = 5; data are representative of two independent experiments. ✝ *p*<0.05 compared with unstimulated BMDCs. (-), unstimulated BMDCs; Curd, curdlan; *Ca*, *C. albicans*; *Ct*, *C. tropicalis*; *Ck*, *C. krusei*.


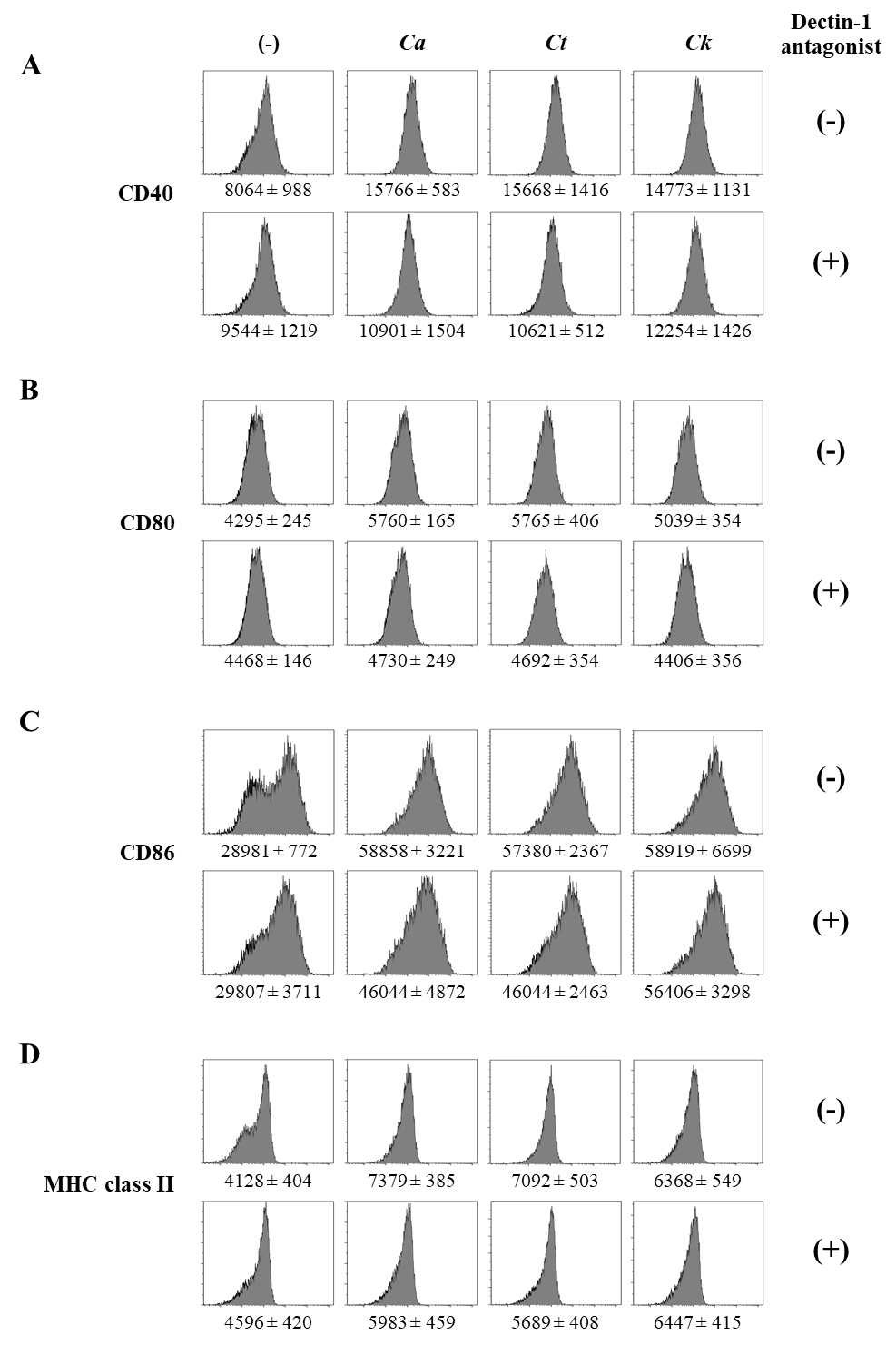


**Supplementary Figure S19**

**Histogram analyses of DC maturation markers at 24 h in dectin-1 blockade assay**

BMDCs were pre-treated with 25 μg/ml of dectin-1 antagonist for 2 h, and the cells were then stimulated with 25 μg/ml of *Candida* β-glucans for 24 h. The expression of DC maturation markers was determined by a flow cytometric analysis. DCs were first identified by gating on CD11c, and the expression of CD40, CD80, CD86 and MHC class II in CD11c^+^ population was subsequently assessed by histogram analyses. The values indicated the MFI (mean ± S.D.). (-), unstimulated BMDCs; *Ca*, *C. albicans*; *Ct*, *C. tropicalis*; *Ck*, *C. krusei*.


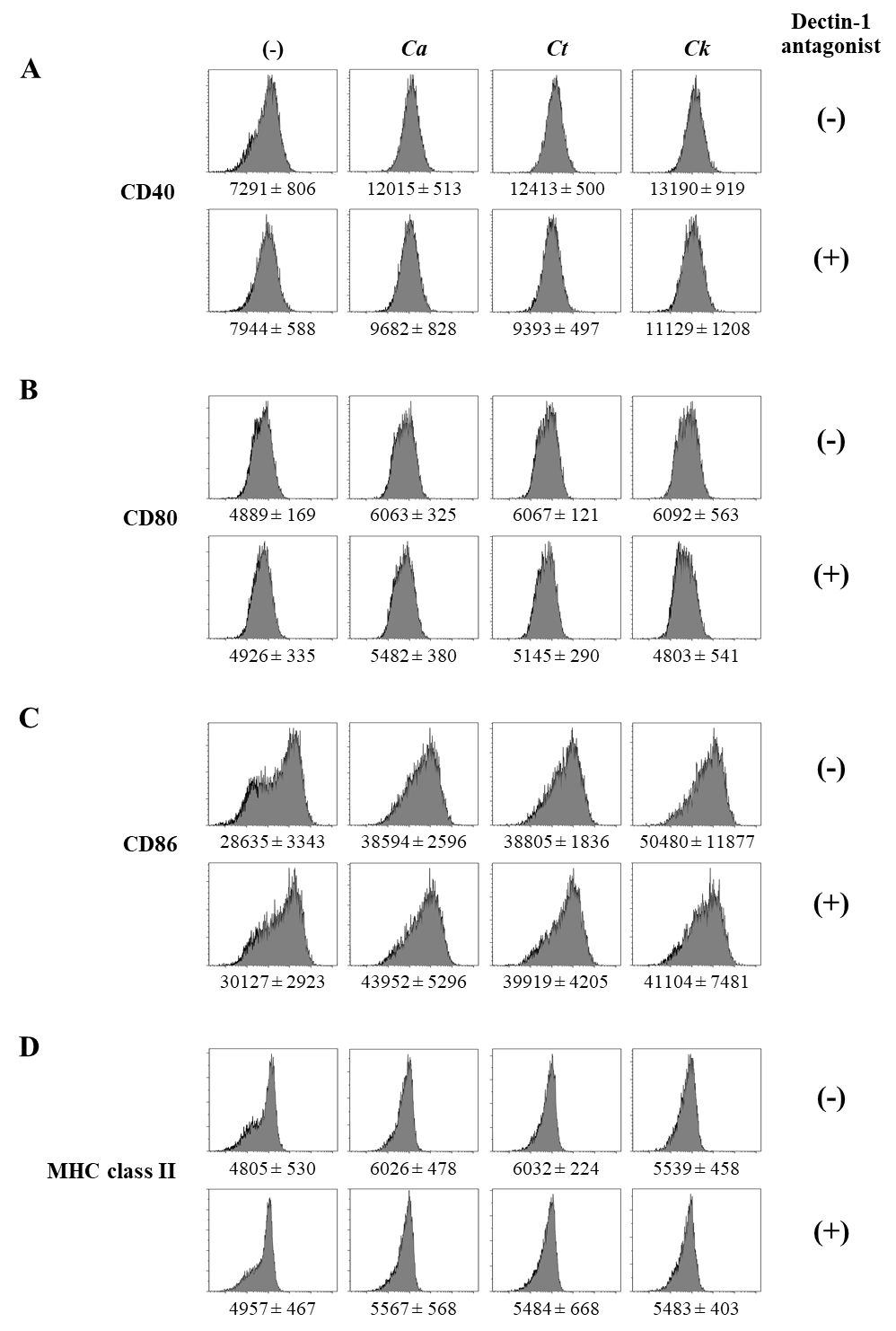


**Supplementary Figure S20**

**Histogram analyses of DC maturation markers at 48 h in dectin-1 blockade assay**

BMDCs were pre-treated with 25 μg/ml of dectin-1 antagonist for 2 h, and the cells were then stimulated with 25 μg/ml of *Candida* β-glucans for 48 h. The expression of DC maturation markers was determined by a flow cytometric analysis. DCs were first identified by gating on CD11c, and the expression of CD40, CD80, CD86 and MHC class II in CD11c^+^ population was subsequently assessed by histogram analyses. The values indicated the MFI (mean ± S.D.). (-), unstimulated BMDCs; *Ca*, *C. albicans*; *Ct*, *C. tropicalis*; *Ck*, *C. krusei*.


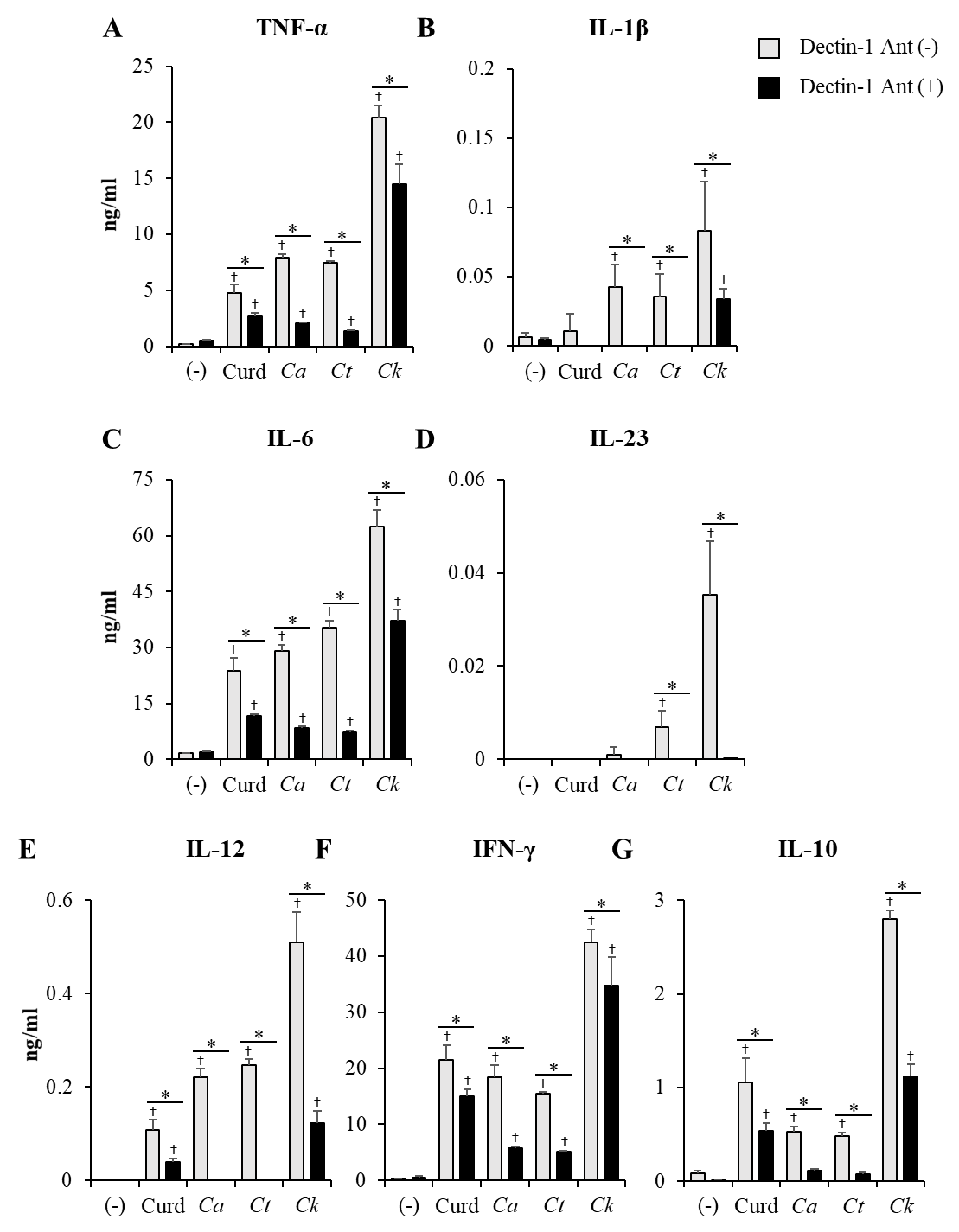


**Supplementary Figure S21**

**Effects of dectin-1 blockade on DC cytokine production (48 h)**

BMDCs were pre-treated with 25 µg/ml of dectin-1 antagonist for 2 h, and then the cells were stimulated with 25 µg/ml of curdlan and β-glucans isolated from *C. albicans*, *C. tropicalis*, and *C. krusei* for 48 h. Levels of **(A)** TNF-α, **(B)** IL-1β, **(C)** IL-6, **(D)** IL-23, **(E)** IL-12, **(F)** IFN-γ, and **(G)** IL-10 were measured in the culture supernatants by ELISA. *n* = 5; Data are representative of two independent experiments. ✝ *p*<0.05 compared with unstimulated BMDCs, * *p*<0.05. (-), unstimulated BMDCs; Curd, curdlan; *Ca*, *C. albicans*; *Ct*, *C. tropicalis*; *Ck*, *C. krusei*.


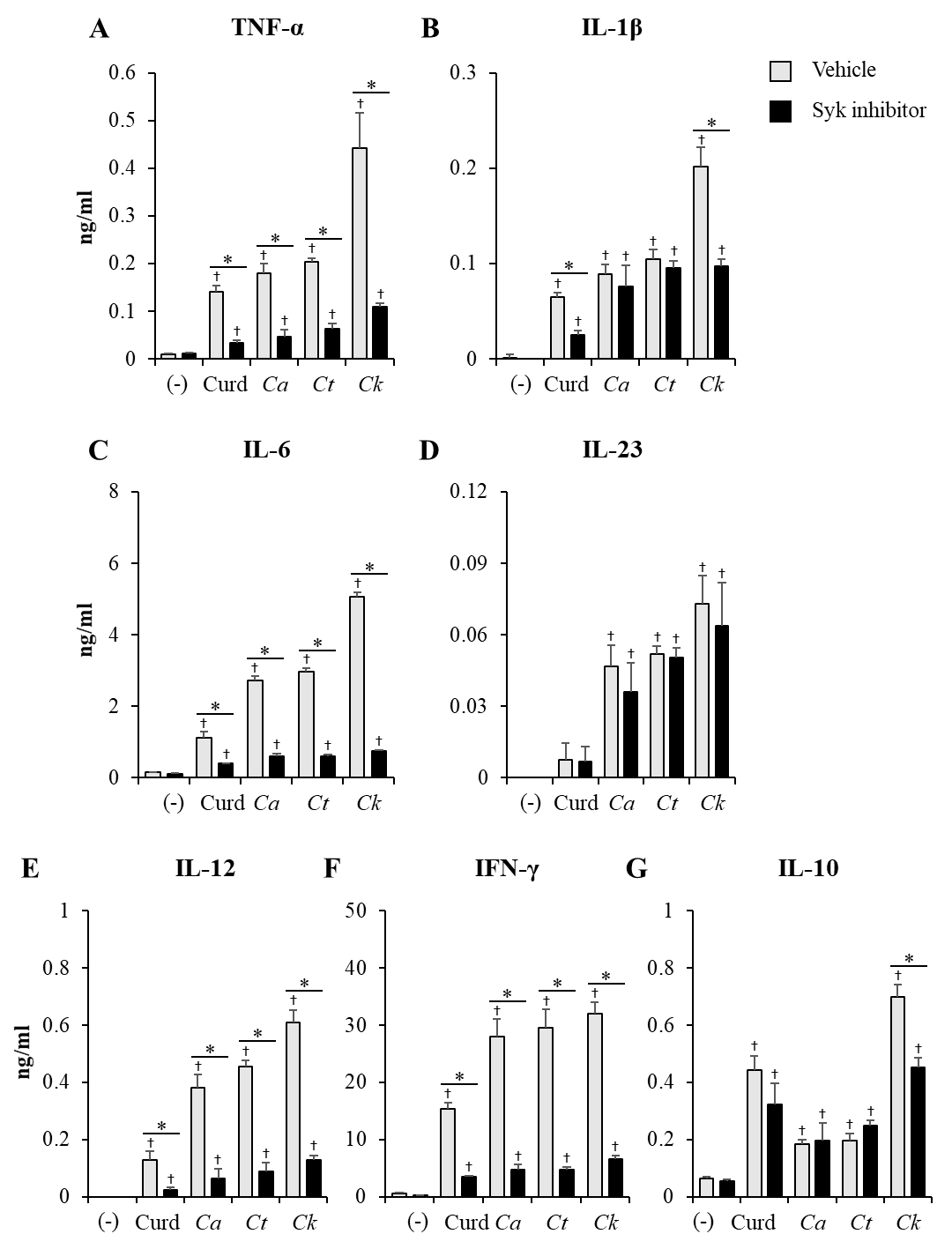


**Supplementary Figure S22**

**Effects of Syk inhibitor on DC cytokine production (24 h)**

BMDCs were pre-treated with 1 µM of Syk inhibitor for 30, and then the cells were stimulated with 25 µg/ml of curdlan and β-glucans isolated from *C. albicans*, *C. tropicalis*, and *C. krusei* for 48 h. Levels of **(A)** TNF-α, **(B)** IL-1β, **(C)** IL-6, **(D)** IL-23, **(E)** IL-12, **(F)** IFN-γ, and **(G)** IL-10 were measured in the culture supernatants by ELISA. *n* = 5; Data are representative of two independent experiments. ✝ *p*<0.05 compared with unstimulated BMDCs, * *p*<0.05. (-), unstimulated BMDCs; Curd, curdlan; *Ca*, *C. albicans*; *Ct*, *C. tropicalis*; *Ck*, *C. krusei*.

**
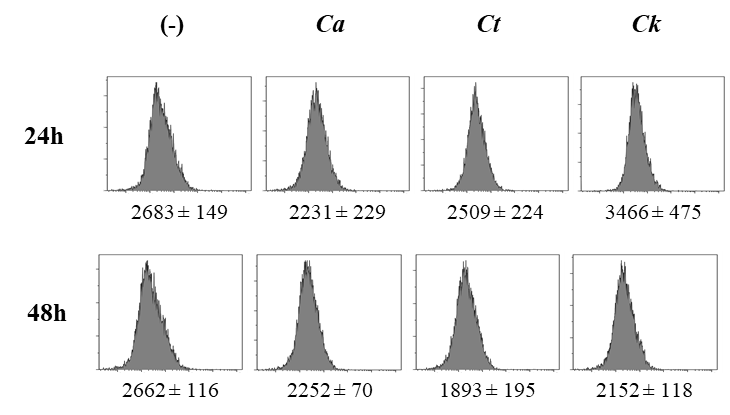
**

**Supplementary Figure S23**

**Histogram analyses of dectin-1 expression on DC**

BMDCs were stimulated with 25 μg/ml of *Candida* β-glucans for 24 h and 48 h. The expression of dectin-1 was determined by a flow cytometric analysis. DCs were first identified by gating on CD11c, and the expression of dectin-1 in CD11c^+^ population was subsequently assessed by histogram analyses. The values indicated the MFI (mean ± S.D.). (-), unstimulated BMDCs; *Ca*, *C. albicans*; *Ct*, *C. tropicalis*; *Ck*, *C. krusei*.

**Supplementary Table 1 Blockade of DC maturation** **by Syk inhibitor**

|  |  | **% Blocking* ± SD** | | | |
| --- | --- | --- | --- | --- | --- |
|  |  | **Curdlan** | ***C. albicans*** | ***C. tropicalis*** | ***C. krusei*** |
| **24 h** | **CD40** | 7.88 ± 4.77 | 4.36 ± 12.37 | 6.55 ± 5.38 | 0.00 ± 1.59 |
|  | **CD80** | 14.36 ± 3.88 | 21.34 ± 8.36 | 21.53 ± 5.36 | 22.11 ± 3.12 |
|  | **CD86** | 37.34 ± 1.89 ^d^ | 34.46 ± 3.16 ^d^ | 36.21 ± 5.87 ^d^ | 27.17 ± 3.11 ^a, b, c^ |
|  | **MHC class II** | 32.60 ± 4.66 | 34.17 ± 7.24 | 22.69 ± 9.47 | 27.31 ± 5.91 |
| **b48 h** | **CD40** | 24.53 ± 3.24 ^b, c^ | 42.02 ± 6.10 ^a, d^ | 41.23 ± 10.42 ^a^ | 22.98 ± 11.41 ^a, b^ |
|  | **CD80** | 18.58 ± 4.37 ^b, c, d^ | 48.37 ± 4.82 ^a^ | 53.72 ± 7.51 ^a^ | 39.72 ± 11.18 ^a^ |
|  | **CD86** | 43.53 ± 3.16 ^b, c^ | 55.82 ± 5.87 ^a^ | 62.11 ± 8.21 ^a, d^ | 43.09 ± 10.93 ^a, c^ |
|  | **MHC class II** | 38.20 ± 2.97 ^d^ | 38.44 ± 8.20 ^d^ | 33.33 ± 3.03 ^d^ | 17.08 ± 6.50 ^a, b, c^ |

*****% Blocking was calculated by the following formula; (average MFI of molecule A expressed on glucan-stimulated DCs – MFI of molecule A expressed on Syk inhibitor treated glucan-stimulated DCs) x 100/average MFI of molecule A expressed on glucan-stimulated DCs). n = 5; Data are representatives of two independent experiments. ^a^*p* < 0.05 compared with Curdlan, ^b^*p* < 0.05 compared with *C. albicans* glucan, ^c^*p* < 0.05 compared with *C. tropicalis* glucan, ^d^*p* < 0.05 compared with *C. krusei* glucan.

**Supplementary Table 2 Blockade of DC cytokine production by Syk inhibitor**

|  |  | **% Blocking* ± SD** | | | |
| --- | --- | --- | --- | --- | --- |
|  |  | **Curdlan** | ***C. albicans*** | ***C. topicalis*** | ***C. krusei*** |
| **24 h** | **TNF-α** | 76.29 ± 3.66 | 73.70 ± 8.18 | 69.34 ± 5.35 | 75.11 ± 1.60 |
|  | **IL-1β** | 61.33 ± 6.88 ^b, c^ | 14.68 ± 24.78 ^a, d^ | 9.39 ± 7.72 ^a, d^ | 51.75 ± 3.59 ^b, c^ |
|  | **IL-6** | 63.77 ± 1.29 ^b, c, d^ | 77.94 ± 2.63 ^a, d^ | 79.39 ± 1.75 ^a, d^ | 85.40 ± 0.49 ^a, b, c^ |
|  | **IL-23** | 7.97 ± 14.31 | 23.30 ± 16.57 | 5.26 ± 7.61 | 12.60 ± 14.58 |
|  | **IL-12** | 80.19 ± 5.06 | 83.13 ± 8.68 | 80.44 ± 6.45 | 79.09 ± 2.66 |
|  | **IFN-γ** | 77.54 ± 0.75 ^b, c^ | 82.86 ± 0.73 ^a, d^ | 83.94 ± 0.87 ^a, d^ | 79.24 ± 0.68 ^b, c^ |
|  | **IL-10** | 27.09 ± 16.12 ^b, c^ | 0.80 ± 10.15 ^a, d^ | 0.00 ± 9.09 ^a, d^ | 35.44 ± 5.01 ^b, c^ |
| **48 h** | **TNF-α** | 72.05 ± 5.24 ^b, c, d^ | 94.13 ± 1.45 ^a^ | 95.81 ± 0.96 ^a^ | 95.63 ± 1.81^a^ |
|  | **IL-1β** | 9.50 ± 17.81 ^b, c^ | 48.20 ± 10.54 ^a, d^ | 58.66 ± 12.14 ^a, d^ | 24.21 ± 11.07 ^b, c^ |
|  | **IL-6** | 73.51 ± 1.14 ^b, c, d^ | 95.14 ± 0.23 ^a^ | 94.89 ± 0.25 ^a^ | 92.54 ± 0.42 ^a^ |
|  | **IL-23** | 74.37 ± 13.81 | 56.81 ± 13.37 | 62.50 ± 12.79 | 68.60 ± 12.71 |
|  | **IL-12** | 50.53 ± 12.97 ^b, c, d^ | 84.15 ± 9.99 ^a^ | 90.27 ± 4.99 ^a^ | 80.89 ± 5.65 ^a^ |
|  | **IFN-γ** | 79.23 ± 1.18 ^b, c, d^ | 96.28 ± 0.29 ^a, d^ | 96.59 ± 0.31 ^a, d^ | 91.28 ± 0.77 ^a, b, c^ |
|  | **IL-10** | 37.88 ± 13.31 ^b, c, d^ | 83.39 ± 3.84 ^a, d^ | 89.45 ± 2.02 ^a, d^ | 72.21 ± 8.24 ^a, b, c^ |

*****% Blocking was calculated by the following formula; (average MFI of cytokine A produced by glucan-stimulated DCs – MFI of cytokine A produced by Syk inhibitor treated glucan-stimulated DCs) x 100/average MFI of cytokine A produced by glucan-stimulated DCs). n = 5; Data are representatives of two independent experiments. ^a^*p* < 0.05 compared with Curdlan, ^b^*p* < 0.05 compared with *C. albicans* glucan, ^c^*p* < 0.05 compared with *C. tropicalis* glucan, ^d^*p* < 0.05 compared with *C. krusei* glucan.
